# Decoding the *Penicillium italicum*–*Citrus* Interaction: Untargeted Metabolomics Sheds Light on a Neglected Postharvest Pathogen

**DOI:** 10.1021/acs.jafc.5c07618

**Published:** 2025-10-16

**Authors:** Evandro Silva, Aline Midori Kanashiro, José Rodrigo Ferreira Maciel, Rodolfo Dantas Lima Junior, Maria Antonia Fraga Botelho, Alana Kelyene Pereira, Stephanie Nemesio da Silva, Jonas Henrique Costa, João Guilherme de Moraes Pontes, Amanda Ferreira da Silva, Igor Dias Jurberg, Roberto G. S. Berlinck, Taicia Pacheco Fill

**Affiliations:** † Instituto de Química, 28132 Universidade Estadual de Campinas, CEP 13083-970 Campinas, São Paulo, Brazil; ‡ Instituto de Química de São Carlos, Universidade de São Paulo, CP 780, CEP 13560-970 São Carlos, São Paulo, Brazil

**Keywords:** *Penicillium italicum*, *citrus*, blue mold, metabolomics, mass spectrometry imaging

## Abstract

*Penicillium italicum*, the causal agent of citrus blue mold, is a major postharvest pathogen that reduces fruit quality and global citrus productivity. Understanding the molecular basis of infection is crucial to reveal virulence mechanisms, host defense responses, and potential targets for disease control. Here, we investigated the metabolic profile of the*Citrus sinensis*–*P. italicum* interaction using mass spectrometry-based metabolomics and desorption electrospray ionization mass spectrometry imaging. Key differential*P. italicum*-derived metabolites were identified, including 12,13-dehydroprolyltryptophanyldiketopiperazine, deoxybrevianamide E, dehydrodeoxybrevianamide E, deoxyisoaustamide, and brevianamide F. To assess its biological role, brevianamide F was chemically synthesized and tested against citrus-associated endophytes. It selectively inhibited*Diaporthe* sp., suggesting that*P. italicum*may utilize this compound as an antimicrobial strategy to modulate the endophytic community during infection. These results provide the first insights into the natural products involved in*P. italicum*association with citrus and point to potential alternative strategies for controlling blue mold disease.

## Graphical abstract

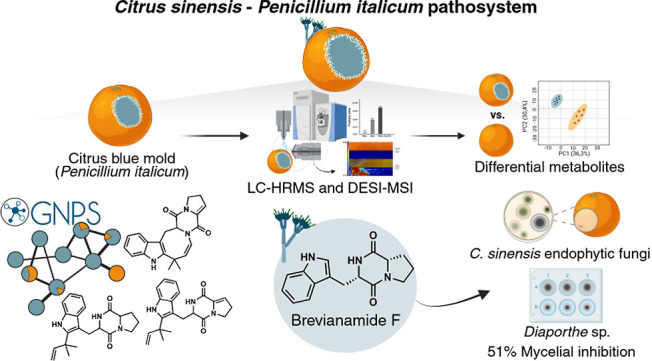

## 1 Introduction

*Citrus*spp. is a fruit crop of great importance for the worldwide economy, with records of total production reaching up to 158 million tons.^1,2^ From this total, sweet orange holds the largest production, around 78.7 million tons.^2,3^ Microbial postharvest diseases pose a major challenge to the citrus industry, accounting for up to 35% of fruit losses^4^ and significantly impacting the global economy. The primary fungal phytopathogens associated with citrus postharvest losses are*Penicillium digitatum*,*Penicillium italicum*, and*Geotrichum citri-aurantii*. These fungi are the causal agents of the green mold, blue mold, and citrus sour rot diseases, respectively.^2,5^ Green mold accounts for up to 90% of total postharvest losses in tropical countries, followed by blue mold and citrus sour rot diseases that undergo slow development in tropical conditions.^6,7^ *P. italicum* is particularly resistant to cold, making the blue mold the most prevalent postharvest disease on fruits stored at low temperatures.^7,8^ Furthermore, blue mold is of great concern due to nesting, a process of fast spreading of the infection on healthy fruits of the same packing box.^1,9^ In China, blue mold is responsible for approximately 33 to 50% losses in annual citrus production.^10^

Blue mold symptoms in citrus peel tissues are characterized by pectin demethylation, swelling of cell wall, plasmolysis, and d-galacturonic acid accumulation during the early stages of infection.^11^ The genome of*P. italicum*has been sequenced, revealing a broader host range compared to*P. digitatum*, with *P. italicum*infecting a variety of fruits, including avocado, mango, melon, and apple.^7,12–14^ Despite the significant negative economic impact of*P. italicum* on fruit crops, the biosynthetic potential of this pathogen has been underexplored, and the virulence factors and infection mechanisms remain unidentified.^7^

Currently, the primary method for controlling blue mold is the use of synthetic fungicides, such as pyrimethanil, imazalil, fludioxonil, and thiabendazole.^3^ However, these chemicals are potentially toxic to both the environment and human health.^3^ In addition, the continuous use of these antifungal agents is increasing fungal resistance, environmental pollution, and alteration of ecological relationships.^15^

Alternative strategies for the control of postharvest diseases have been studied, including the application of electromagnetic and ionization radiations,^16,17^ the use of essential oils and plant extracts with antifungal proprieties,^18,19^ application of nanotechnology,^20^ and application of organic and inorganic salts as well as the use of biocontrol agents.^5,21^ However, none of these methods have demonstrated the same efficiency as the application of synthetic fungicides.^15,22^

A better understanding of the biochemical pathways involved in pathogen–host interactions, as well as the identification of metabolites produced during infection, could lead to the discovery of novel virulence factors. This, in turn, could pave the way for developing specific and environmentally friendly control strategies for fungal plant diseases.^23,24^ To date, no*P. italicum* secondary metabolites have been associated with the infection process on citrus hosts. While some studies reported a few metabolites produced in the in vitro cultures of*P. italicum*, it remains unclear whether these metabolites play a role in the interaction with the citrus hosts.^7^

To address these knowledge gaps, we conducted an untargeted metabolomics study on blue mold disease caused by*P. italicum* in*Citrus sinensis*. This study applied ultrahigh-performance liquid chromatography coupled with high-resolution mass spectrometry (LC-HRMS) and desorption electrospray ionization mass spectrometry (DESI-MSI) for the first time to investigate the blue mold disease. The combination of these techniques allowed us to investigate the secondary metabolites produced by the pathogen during infection and to monitor the metabolic defense strategies of*Citrus sinensis*. Biological assays were also carried out to further explore the ecological roles of fungal secondary metabolites in blue mold disease.

## 2 Material and Methods

### 2.1 Fungal Strain

*P. italicum* fungal strain was deposited in the Spanish Collection of Type Cultures (CECT) under the access code CECT20909. Potato dextrose agar (PDA, Acumedia) was used to maintain the strain. The medium was autoclaved at 103 kPa (121 °C) for 15 min and then transferred to Petri dishes for inoculation of the fungus, which was incubated in biochemical oxygen demand for 7 days at 23 °C in the dark. The conidia suspension was then obtained in sterile water at a final concentration of 10^6^ conidia/mL using a Neubauer chamber.

### 2.2 Fruit Inoculation with*P. italicum* and Sample Collection

Sweet oranges (*Citrus sinensis*) of similar size, color, and apparent maturity were purchased on the same day from a local grocery store (Campinas, SP, Brazil). Fruits were processed within 24 h of purchase and kept at ambient laboratory conditions (21 ± 1 °C, 60–70% relative humidity) until inoculation to minimize variation due to postharvest handling. All fruits were surface-sterilized by immersion in a 2% (v/v) NaClO solution for 5 min, rinsed thoroughly with distilled water, and air-dried at 25 °C. Small incisions of approximately 1 cm^2^ were made on the surface of each orange, and 5 μL of a *P. italicum* conidial suspension (10^6^ conidia/mL) was applied to each wound site. The inoculated fruits were placed individually in sterile 500 mL beakers and incubated for 10 days at 21 °C. Control samples were prepared under identical conditions. Ten fruits were used for each group (infected and control). After the incubation period, peel sections (4 cm × 4 cm) surrounding the infection sites were excised, transferred to sterile crucibles, and ground into a fine powder using liquid nitrogen. The powdered samples were stored at −20 °C until further extraction.

### 2.3 Metabolite Extraction and Untargeted Metabolomics Analysis

Metabolites were extracted by the addition of 1 mL of MeOH into 100 mg of each orange sample (controls and infected oranges) and then left for 25 min in an ultrasound bath. The extracts were centrifuged at 12,000 rpm for 10 min at 4 °C, and then the supernatants were collected, dried under N_2_ flow, and resuspended in 1 mL of MeOH. The resuspended extracts were filtered through 0.22 μm PTFE filters into 2 mL vials. Quality control (QC) samples were prepared by gathering 10 μL of each sample in a single vial.

The samples were analyzed using a Thermo Fisher RSLCnano U3000 UHPLC instrument coupled to a Q-Exactive Orbitrap-MS spectrometer (Thermo Fisher Scientific, USA) equipped with a heated electrospray ionization source. Chromatographic separation was performed with an Intensity Solo C18 2.2 μm column (2.1 mm × 100 mm, Bruker Daltonics, Bremen, Germany). Mobile phases were 0.1% (v/v) formic acid in H_2_O (A) and 0.1% formic acid in MeCN (B), 40 °C for elution temperature, and 250 μL/min for the flow rate. The elution gradient consisted of 5% B (0–5 min), 40% B (5–10 min), 70% B (10–14 min), 80% B (14–15 min), 98% B (15–20 min), and 5% B (21–24 min). Mass spectra acquisition was performed using the following parameters: electrospray ionization in positive mode, capillary voltage: +3.5 kV, capillary temperature: 250 °C, S-lens of 50 V and *m*/*z* range 100–1500. LC–MS spectra were recorded using a normalized collision energy of 30 eV, and 5 precursors per cycle were selected (data-dependent acquisition mode).

### 2.4 Data Processing and Statistical Analyses

Raw data files (.raw) were converted to the .mzXML format using MSConvert.^25^ The converted files were processed using MZMine3 (version 3.9.0). The parameters used during processing were as follows: in the UHPLC section, smoothing was applied (true), stable ionization across samples (true), crop retention time (RT) (0.30–20.00 min), maximum peaks in the chromatogram (15), minimum consecutive scans (4), approximate feature full width at half-maximum (0.080 min), RT tolerance (intrasample) (0.040 min), and RT tolerance (sample-to-sample) (0.100 min). For Orbitrap parameters, the following were applied: ion mode (positive), absolute intensity, MS1 noise level (5.0 × 10^5^), MS2-MSn noise level (6.0 × 10^3^), minimum feature height (1.0 × 10^6^), *m*/*z* tolerance (scan-to-scan) (5.0 ppm), *m*/*z* tolerance (within-sample) (3.0 ppm), and *m*/*z* tolerance (sample-to-sample) (5.0 ppm). In filters, the original feature list was removed, and one sample was used as the maximum number of samples per aligned feature. Finally, solvent blank features were filtered out from the final feature list.

To perform multivariate analysis, the processed data file in the .csv format was exported to MetaboAnalyst 5.0 (www.metaboanalyst.ca). The features were normalized by sum and scaled by using the Pareto scaling method. Principal component analysis (PCA) was conducted to identify differences in metabolic profiles between control and*P. italicum*-infected oranges. To investigate altered metabolites between groups, the supervised partial least-squares discriminant analysis (PLS-DA) method was applied. Differentially expressed metabolites were identified based on the values of the variable importance in the projection (VIP) ≥ 1 obtained from the PLS-DA model.

### 2.5 Molecular Networking and Metabolite Annotation

After data processing from MZMine3 (version 3.9.0), two main files were exported: a .csv table containing the quantification of the detected features and an .mgf file with MS/MS spectra corresponding to the precursor ions. These files were submitted for analysis on the Global Natural Products Social Molecular Networking (GNPS) platform (https://gnps.ucsd.edu),^26^ with the aim of annotating the features present in the samples and constructing molecular networks based on fragmentation spectrum similarity. The analysis was carried out using the feature-based molecular networking workflow to annotate features and construct molecular networks based on the similarity of MS/MS fragmentation spectra, enabling evaluation of the chemical composition of infected and control orange samples.

The general parameters included precursor ion mass tolerance (0.01 Da) and fragment ion tolerance (0.02 Da). For networking construction, the minimum cosine for clustering nodes (0.70) in a molecular family was set, and the minimum match of peaks between the nodes was set as a requirement to compose node (4). About library search, annotations were used with the library minimum cosine (0.80) and library minimum match peaks (4). The resulting molecular network was visualized using Cytoscape software (version 3.7.2, Cytoscape Consortium, San Diego, CA, USA),^27^ with each node corresponding to specific *m*/*z* and RT ions (feature). Edges represent cosine similarity scores calculated between nodes based on fragmentation spectra (MS/MS). An authentic standard was chemically synthesized according to the methodology described at Kieffer et al.^28^ and used to achieve level-1 identification of brevianamide F based on RT and MS/MS data comparison. The bar charts were constructed using GraphPad Prism version 8.

### 2.6 In Vitro Secondary Metabolites Produced by *P. italicum*

*P. italicum* (10^6^ conidia/mL) was inoculated into three different liquid culture media: orange serum broth (HiMedia Laboratories GmbH, Odenwald, Germany), potato dextrose broth (PDB, Difco-BD Diagnostics, Sparks, MD, USA), and glucose yeast peptone medium. The liquid cultures (50 mL) were prepared in triplicate and incubated for 7 days under static conditions at 23 °C in the dark.

The aqueous phase (50 mL) was transferred into a separation funnel, followed by the addition of 100 mL of EtOAc. After promoting contact between the two phases, the system was allowed to stand for 10 min to achieve complete phase separation. The organic phase was then carefully collected, and the residual water was dried with anhydrous magnesium sulfate. The resulting extracts were concentrated under a N_2_ flow and resuspended in 1 mL of MeOH for subsequent LC–HRMS analyses. The LC-HRMS analyses were performed as described in Section 2.5.

### 2.7 Mass Spectrometry Imaging Analyses

Mass spectrometry imaging (MSI) analyses were performed directly on the flavedo surface of orange peel samples infected with*P. italicum* at 6 days postinoculation and control samples. Two sample preparation procedures were tested: direct analysis of the infected orange peel and the imprinting technique. For the imprinting method, orange peels were uniformly pressed on a silica plate. For analyses performed directly from the infected peel, the orange peel was pressed against a modeling clay containing superglue, ensuring a plane surface.

The analyses were performed in a Thermo Scientific Q-Exactive MSI using a DESI source (Omni Spray 2D-3201 model, Prosolia, Indianapolis, USA) configured with a height of 2.5 mm for the emitter, an inlet height of the mass spectrometer at 0.1 mm, the inlet for an emitter distance of 3.8 mm, and a spray angle of 58° with a voltage of 5.0 kV. Other parameters adjusted were the capillary temperature at 320 °C, S-lens at 100 V, and pressure of the ultrapure nebulizing gas at 160 psi with a flow rate of 10.0 μL/min. Images were acquired in a *m*/*z* range between 100 and 1.500 with a step size of 200 μm, scan rate of 741 μm/s, and pixel size of 200 μm × 200 μm. BioMap software (Novartis Institutes for BioMedical Research) was used for image data processing, and Xcalibur software (Thermo Fisher Scientific) was used for LC–MS data processing.

### 2.8 Isolation and Identification of Endophytic Fungi from*Citrus sinensis*

Endophytic fungi were isolated from*Citrus sinensis* fruits as previously described by Godinho et al.^29^ After washing the fruits with running water, the epiphytic micropopulation was eliminated by immersing the orange peel fragments in 70% EtOH for 2 min, followed by immersion in a solution of 2.5% sodium hypochlorite for 2 min. Then, the fragments were washed with sterile distilled water three times consecutively. Using sterile tweezers, three 0.5 cm fragments were placed in a Petri dish previously prepared with PDA culture medium and left for 7 days at 25 °C. The mycelia that emerged from the peel tissues were aseptically extracted in a laminar flow hood, and fungal fragments were seeded in individual Petri dishes with PDA medium.

Identification of the endophytic fungal strains was carried out from fungal cultures grown on PDA at 28 °C, and genomic DNA was extracted and purified using a phenol/chloroform protocol.^30^ After DNA purification, the internal transcribed spacer (ITS) region was amplified by using the ITS1/ITS4 primer pair. The resulting PCR product was purified using the GFX PCR DNA and Gel Band Purification Kit (GE Healthcare) and directly sequenced with an ABI 3500XL Series automatic sequencer (Applied Biosystems). The consensus sequence was compared with the sequences of organisms in the GenBank databases and from the Fungal Biodiversity Centre (CBS). The DNA sequences were aligned using CLUSTAL X software.^31^ Phylogenetic analyses were performed using MEGA 6.0 software.^32^ The distance matrix was calculated according to the Kimura model.^33^ The construction of the dendrogram from the genetic distances was carried out using the Neighbor-Joining method,^34^ with bootstrap values calculated by 1000 resamples, using the MEGA 6.0 software.

### 2.9 Coculture Growth Conditions and Extraction of Secondary Metabolites

In vitro cocultures of *P. italicum* with the endophytic fungi*Diaporthe* sp. and *Colletotrichum* sp., both endophytes isolated from*Citrus sinensis*, were prepared in Petri dishes containing 25 mL of PDA. A volume of 10 μL of the pathogen and each endophyte fungal spore suspension (10^6^ spores mL^–1^) were inoculated on opposite sides of Petri dishes. The plates were incubated in the dark at 25 °C for 7 days. Experiments were conducted in triplicate.

To extract fungal cocultures, the zone of confrontation between each fungal pair and the monoculture region was carefully cut and transferred to a centrifuge tube. Then, 1 mL of MeOH was added to each centrifuge tube. Tubes were sonicated for 45 min in an ultrasonic bath, followed by centrifugation at 12,000 rpm for 10 min. The resulting extract was resuspended in 1 mL of MeOH, filtered on a 0.22 μm PTFE filter for further LC-HRMS analysis.

### 2.10 Antifungal Assays

The antifungal assay of brevianamide F against*Diaporthe* sp. and*Colletotrichum* sp., using a microbroth dilution assay, was conducted following the recommendations of the Clinical and Laboratory Standards Institute—CLSbI (2008), with slight modifications. A stock solution of brevianamide F at a concentration of 1 mg/mL was prepared in ethanol/water (1:1, v/v). This stock was then diluted in PDA medium to final concentrations of 0.05, 0.10, and 0.30 mg/mL. Imazalil was used as a positive control at the same concentration. A solution of ethanol/water (1:1, v/v) was used as the negative control. Then, 4 mL of each diluted solution was transferred to a 6-well microplate. A plug of *Diaporthe*sp. fungus and 10 μL of a spore solution containing 10^6^ spores mL^–1^ of *Colletotrichum* sp. were inoculated into each well. The microplates were then incubated in the dark at 25 °C for 96 h. The assays were conducted in triplicate.

### 2.11 Confocal Microscopy Analysis of *Diaporthe*sp. Growth in Response to Brevianamide F

To evaluate the influence of brevianamide F on the growth of*Citrus sinensis* endophytic fungi, confocal microscopy analysis was performed. In this experiment, 10 μL of *Diaporthe* sp. spore suspension (10^6^ spores mL^–1^) was inoculated onto sterile microscope slides placed in Petri dishes containing 15 mL of PDA supplemented with brevianamide F at a concentration of 0.3 mg/mL. The assays were conducted in triplicate. As a control, a 10 μL aliquot of a *Diaporthe* sp. spore suspension (10^6^ spores mL^–1^) was inoculated onto sterile microscope slides in Petri dishes containing PDA. The plates were then incubated in a dark environment at 25 °C for 96 h. Following the incubation period, the microscope slides were carefully removed from the Petri dish. The samples were then stained with Congo Red (0.25% w/v in H_2_O) for 20 min and subsequently washed with distilled H_2_O. The analysis was performed using a Leica TCS SP5 microscope. Excitation was achieved using the 543 nm emission line of a He–Ne laser, and light ranging from 570 to 680 nm was collected for analysis.^35^

### 2.12 Isolation, Preparation, and Analysis of Marfey’s Derivatives Reaction

Brevianamide F was purified from the crude extract of *P. italicum,* and its configuration was evaluated using Marfey’s reaction. For this, the strain was inoculated in 50 Petri dishes containing PDA and incubated at 25 °C for 7 days. The contents of the Petri dishes were then cut into 1 cm^2^ fragments, transferred to 500 mL Erlenmeyer flasks, and extracted with ethyl acetate (1:1 m/v) under agitation at 200 rpm for 1 h. The organic phase was separated, concentrated under reduced pressure, and subjected to semipreparative HPLC (Shimadzu LC-10AD) with a C18 column (Phenomenex Luna 250 × 10 mm, 5 μm, USA). Gradient elution was performed with water/acetonitrile (0.1% formic acid) increasing from 10% to 100% acetonitrile over 30 min at a 3.8 mL/min flow rate. Fractions were analyzed by HPLC-UV, pooled, concentrated, and characterized by ^1^H NMR and HRMS to confirm the chemical structure.

Brevianamide F (0.2 mg) was hydrolyzed for 24 h in 6.0 M HCl at 50 °C. After being cooled, the solution was evaporated to dryness and redissolved in H_2_O (50 μL). The hydrolysis solution was derivatized by adding 200 μL of 0.5% (w/v) FDAA (Marfey’s reagent; 1-fluoro-2,4-dinitrophenyl-5-l-alanine amide) in acetone solution. Subsequently, 20 μL of a 1 M NaHCO_3_ solution was added, and the mixture was incubated at 45 °C for 40 min. The reaction was quenched by adding 20 μL of 2 M HCl. The solvents were evaporated to dryness, and the resulting residues were dissolved in 20 μL of MeOH.^36^ Separately, standards of l-tryptophan, d-tryptophan, l-proline, and d-proline were derivatized with FDAA in the same manner as the natural product.

The FDAA-derived products were analyzed by analytical HPLC (SHIMADZU model 2540) equipped with a PDA detector using a C18 column (Phenomenex Luna 250 × 4.6 mm, 5 μm, USA) with a flow rate of 1.0 mL/min. Separation was performed using an isocratic gradient of 40:60% (A/B), where (A) H_2_O with 0.1% formic acid and (B) MeOH, for 20 min. The retention times of the FDAA derivatives of l-tryptophan and l-proline were determined and compared with those of the reaction product of Brevianamide F with FDAA. Detection was performed at 254 nm. The retention times of the FDAA derivatives of l-tryptophan and l-proline were determined and compared with those of the corresponding synthetic standards.

## 3 Results

### 3.1 Blue Mold Disease Symptoms and LC–HRMS Analysis of Blue Mold Disease

The oranges used in this study were successfully inoculated with*P. italicum*. At 10 days post-inoculation, symptoms of the blue mold disease were clearly visible (Figure 1), with approximately 40% of the fruit infected, exhibiting characteristic blue sporulation. In contrast, the control fruits remained symptomless. LC-HRMS analyses of the infected oranges at 10 days post-inoculation and the controls revealed distinct metabolic profiles, highlighting infection-associated metabolic changes at this time point (Figure S1).

**Figure 1:**
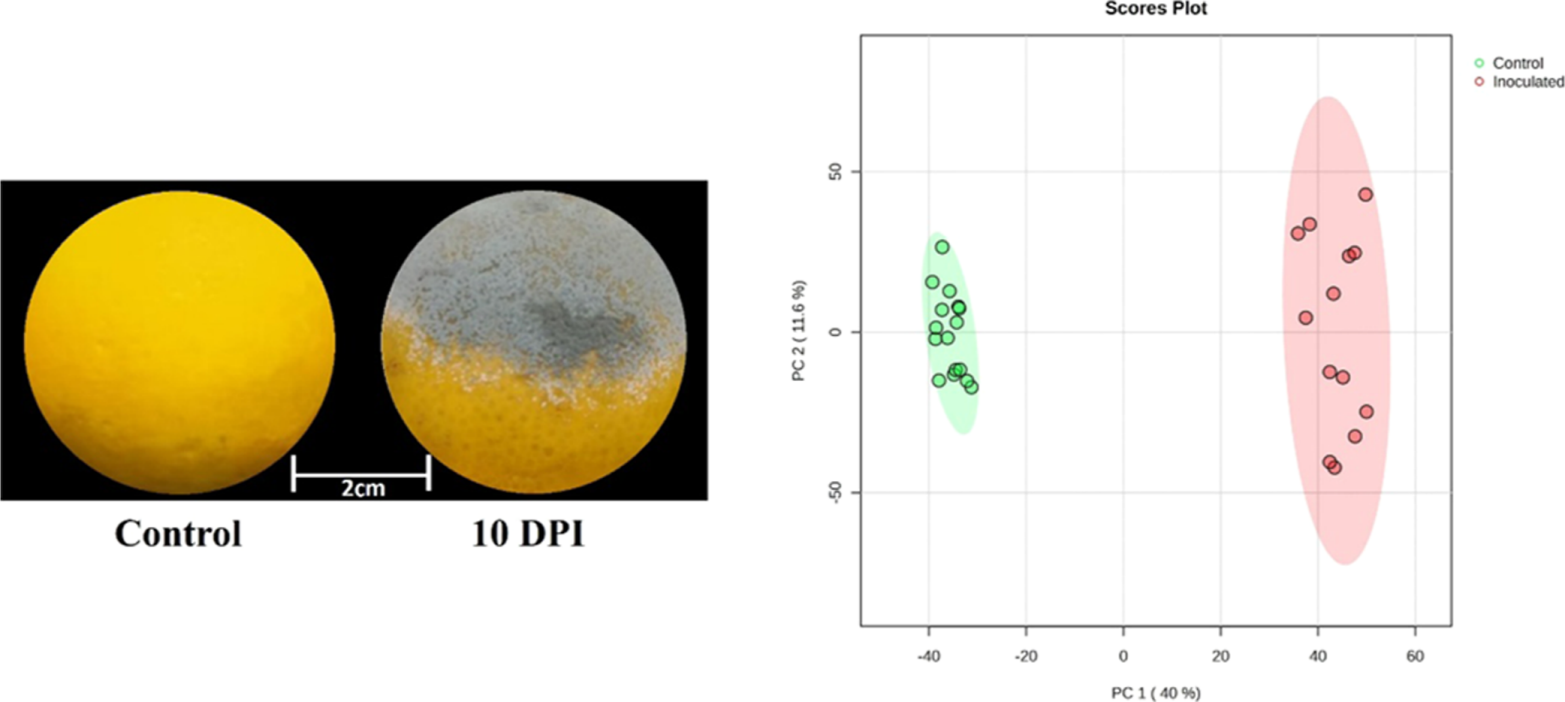
Metabolic changes in oranges infected with *P. italicum* at 10 days post-inoculation. (Left) Representative images of healthy (control) and*P. italicum*-infected oranges at 10 days post-inoculation, showing extensive fungal colonization. Scale: 2 cm. (Right) PCA of metabolic profiles from control (green) and infected (red) fruit samples at 10 days post-inoculation. A clear separation is observed between the two groups, with PC1 accounting for 40.0% and PC2 for 11.6% of the total variance, indicating significant infection-induced metabolic reprogramming.

### 3.2 Multivariate Data Analysis

To evaluate metabolic differences between*P. italicum*-infected and non-infected oranges, we performed PCA (Figure 1). The results revealed a clear separation between the control and infected groups, indicating substantial metabolic alterations induced by the infection. Principal component 1 (PC1) accounted for 40% of the total variance and primarily distinguished the two groups, with infected samples clustered at positive PC1 values and control samples clustered at negative values. Principal component 2 (PC2) explained 11.6% of the variance. All samples were contained within their respective 95% confidence ellipses, supporting the robustness of the observed separation.

Furthermore, the PCA plot confirmed the stability and reproducibility of the LC-HRMS analysis, as evidenced by the clustering of the QC samples, which were clearly separated from the experimental samples (Figure S2). These findings indicate substantial alteration in the fruit metabolome upon*P. italicum* infection, directly reflecting the metabolic changes associated with the infection process (Figure 1).

Additionally, supervised multivariate statistical analysis using the PLS-DA model was performed to identify the key features responsible for discriminating between the groups of interest. A classification model with the two groups (control and infected orange samples) was selected to assess the main features driving the final classification within the samples. The score plot (Figure S2) clearly illustrates the separation of the groups using the two most significant components of the PLS-DA model. Furthermore, the variable importance on the projection (VIP) plot (Table 1) highlights the features that most contributed to the accurate classification of the samples based on the PLS-DA coefficients.

**Table 1:** Secondary Metabolites Annotated in the*Citrus sinensis*–*P. italicum* Pathosystem and in*P. italicum* In Vitro Samples^a^

ID	scan	metabolite annotation	molecular formula	[M + H]^+^ theoretical mass (*m*/*z*)	[M + H]^+^ measured mass (*m*/*z*)	mass accuracy (ppm)	in vivo control inoculated		in vitro	VIP	GNPS library accession
1	558	asparagine	C_4_H_8_N_2_O_3_	133.0608	133.0607	–0.08	X	X	n.d	1.00	CCMSLIB00006120683
2	1534	4-hydroxycinnamyl alcohol	C_9_H_10_O_2_	151.0754	151.0753	–0.07	X	X	n.d	1.51	supplementary data
3	836	phenylalanine	C_9_H_11_NO_2_	166.0863	166.0862	–0.06	X	X	n.d		CCMSLIB00003135371
4	687	synephrine	C_9_H_13_NO_2_	168.1019	168.1018	–0.06	X	X	n.d		CCMSLIB00004691344
5	2371	indolelactic acid	C_11_H_11_NO_3_	206.0811	206.0810	–0.05	X	X	n.d	1.50	CCMSLIB00006684092
6	6090	nootkatone	C_15_H_22_O	219.1743	219.1742	–0.05	X	X	n.d		CCMSLIB00005763621
7	1081	feruloyl putrescine	C_14_H_20_N_2_O_3_	265.1547	265.1546	–0.04	X	X	n.d		CCMSLIB00005748443
8	1356	3′,5,7-trihydroxyflavanone	C_15_H_12_O_5_	273.0757	273.0755	–0.07	X	X	n.d		CCMSLIB00006411935
9	3778	naringenin	C_15_H_12_O_5_	273.0757	273.0756	–0.04	X	X	n.d	1.46	CCMSLIB00010105222
10	3978	hesperetin	C_16_H_14_O_6_	303.0850	303.0861	0.36	X	X	n.d	1.48	CCMSLIB00006374511
11	6944	tetramethyl-*O*-scutellarein	C_19_H_18_O_6_	343.1176	343.1173	–0.09	X	X	n.d		CCMSLIB00006422351
12	5337	tangeretin	C_20_H_20_O_7_	373.1282	373.1281	–0.03	X	X	n.d	1.36	CCMSLIB00012320217
13	4959	nobiletin	C_21_H_22_O_8_	403.1387	403.1386	–0.02	X	X	n.d	1.45	CCMSLIB00012349534
14	2719	hesperetin 7-*O*-glucoside	C_22_H_24_O_8_	465.1324	465.1389	1.40	X	X	n.d		supplementary data
15	2248	diosmin	C_28_H_32_O_15_	609.1814	609.1810	–0.07	X	X	n.d	1.37	CCMSLIB00012176442
16	2410	hesperidin	C_28_H_35_O_15_	611.1970	611.1966	–0.07	X	X	n.d	1.46	CCMSLIB00012176443
17	2215	12,13-dehydroprolyltryptophanyldiketopiperazin	C_16_H_15_N_3_O_2_	282.1237	282.1236	–0.04	n.d	X	X	1.49	supplementary data
18	4265	brevianamide F	C_16_H_17_N_3_O_2_	284.1394	284.1392	–0.07	n.d	X	X	1.44	
19	4570	deoxyisoaustamide	C_21_H_21_N_3_O_2_	348.1706	348.1707	0.03	n.d	X	X	1.38	CCMSLIB00012438440
20	4280	dehydrodeoxybrevianamide E	C_21_H_23_N_3_O_2_	350.1863	350.1862	–0.03	n.d	X	X	1.50	supplementary data
21	4506	deoxybrevianamide E	C_21_H_25_N_3_O_2_	352.2020	352.2013	–0.20	n.d	X	X	1.34	supplementary data
22	4012	brevianamide A	C_21_H_23_N_3_O_3_	366.1817	366.1808	–0.25	n.d	X	X	1.03	supplementary data

a Differentially expressed metabolites with VIP values ≥1 obtained from the PLS-DA model; X: detected, n.d: not detected.

### 3.3 Molecular Networking of the*P. italicum*–*Citrus* Interaction

Molecular networking is a bioinformatics approach that, when integrated into a metabolomics workflow, enables the visualization of molecular groupings based on similarities in the MS/MS fragmentation patterns, known as spectral families. This method facilitates the comparison of chemical profiles by linking experimental spectra to reference spectra through spectral similarity. Molecular networks are generated by aligning MS/MS spectra using a cosine scoring algorithm, where scores range from 0 to 1, with values closer to 1 indicating higher spectral similarity and greater confidence in metabolite identity.^26^

In this study, a molecular network was constructed based on the metabolic profile of orange fruits infected with*P. italicum* at 10 days post-inoculation. The resulting network is shown in Figure S3, which highlights the overall molecular organization derived from the MS/MS data. Metabolite annotation was performed by using the GNPS library and complementary natural product databases. A total of 22 secondary metabolites were putatively annotated (Table 1) in both control and inoculated groups, with some belonging to citrus-specific spectral families, such as hesperetin 7-*O*-glucoside, naringenin, 3′,5,7-trihydroxyflavanone, and diosmin. Among these, diosmin was highlighted as a differential metabolite, showing VIP values ≥1 in the PLS-DA model (Table 1). These metabolites are known to play a crucial role in the defense mechanisms of citrus plants, reinforcing previous findings.^24^

Among the annotated compounds, a distinct molecular family consisting of diketopiperazine alkaloids was observed and is shown in Figure 2. This cluster includes metabolites exclusively detected in the infected orange samples at 10 days post-inoculation, reinforcing their association with the pathogenic activity of*P. italicum*. Six diketopiperazine alkaloids were identified in this group (Table 1), all sharing similar fragmentation patterns. Importantly, all six compounds were statistically significant differential metabolites in the multivariate analyses and exhibited VIP scores ≥1, further supporting their relevance in the host–pathogen interaction (Table 1).

**Figure 2:**
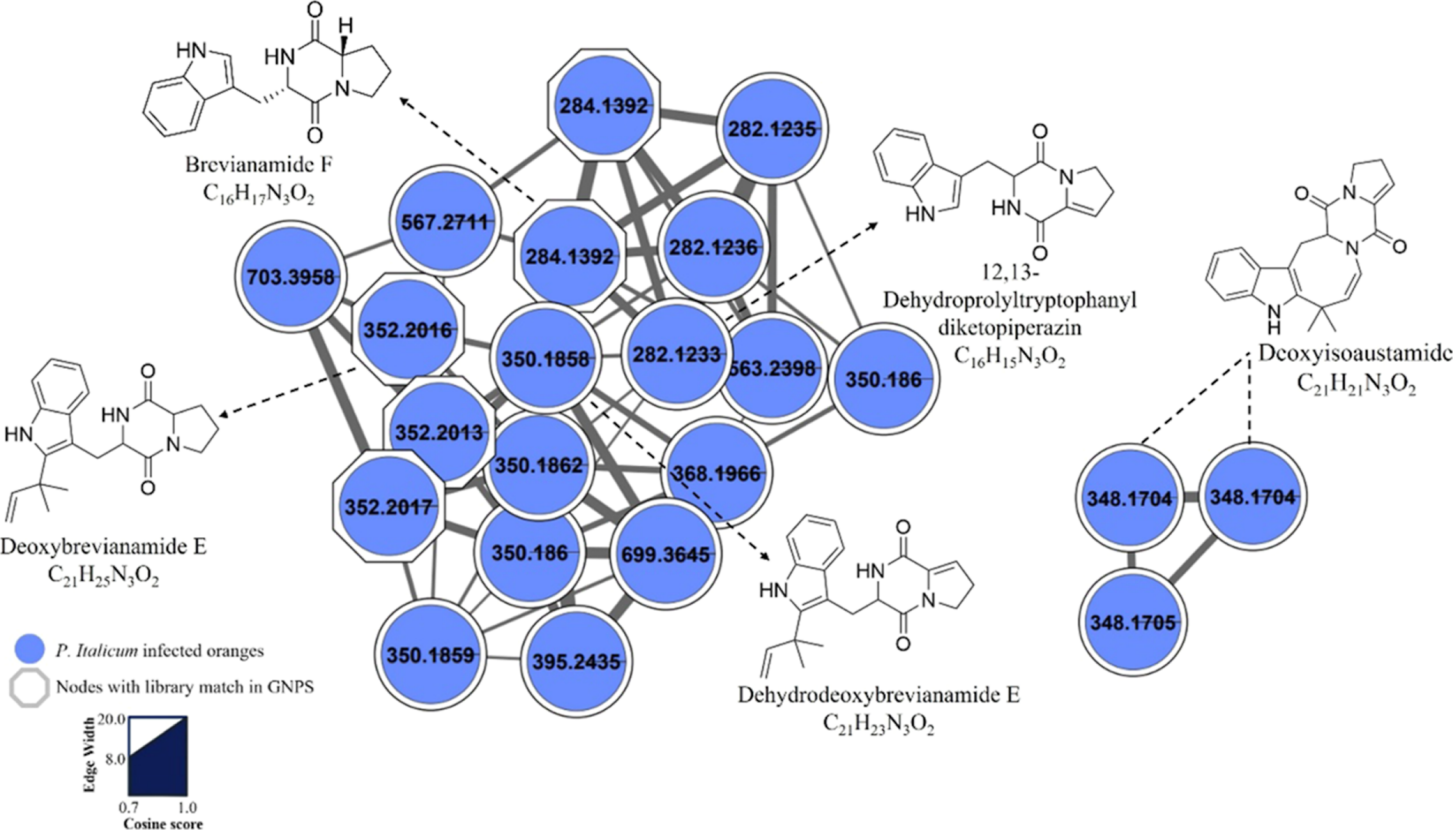
Molecular network cluster of diketopiperazine alkaloids identified in*P. italicum*-infected oranges. Blue nodes represent metabolites exclusively detected in infected samples. Nodes with a polygonal shape indicate spectral matches with GNPS library compounds. Several interconnected nodes form a cluster with high cosine similarity, suggesting structurally related diketopiperazines. Annotated compounds include brevianamide F, deoxybrevianamide E, dehydrodeoxybrevianamide E, deoxyisoaustamide, and 12,13-dehydroprolyltryptophanyldiketopiperazine. This cluster highlights the metabolic specialization associated with*P. italicum* infection. Edge thickness corresponds to the cosine score, with thicker edges indicating higher spectral similarity.

### 3.4 In Vivo Production and Spatial Mapping of Secondary Metabolites during*P. italicum* Infection

In addition to conducting untargeted metabolomics using LC-HRMS, we introduced for the first time the MSI technique in this pathosystem. Mass spectrometry imaging (DESI-MSI) was employed to spatially map compounds in infected citrus tissues, particularly those detected by LC-HRMS (Figure 2), which were exclusively found in the infected orange samples. Our MSI analysis revealed the accumulation of the same diketopiperazine alkaloids (Figure 3). The images showed that both brevianamide F (Table 1, compound 18) with [M + H]^+^ *m*/*z* 284.1394 (Table 1), and deoxyisoaustamide (Table 1, compound 19) with [M + H]^+^ *m*/*z* 348.1707 (Table 1), exhibited a localized distribution in the area of active infection, suggesting that their production are potentially associated with the colonization of host tissue (Figure 3). No signals were detected in the corresponding regions of uninfected fruits, confirming the specific production of these metabolites by the fungus during infection.

**Figure 3:**
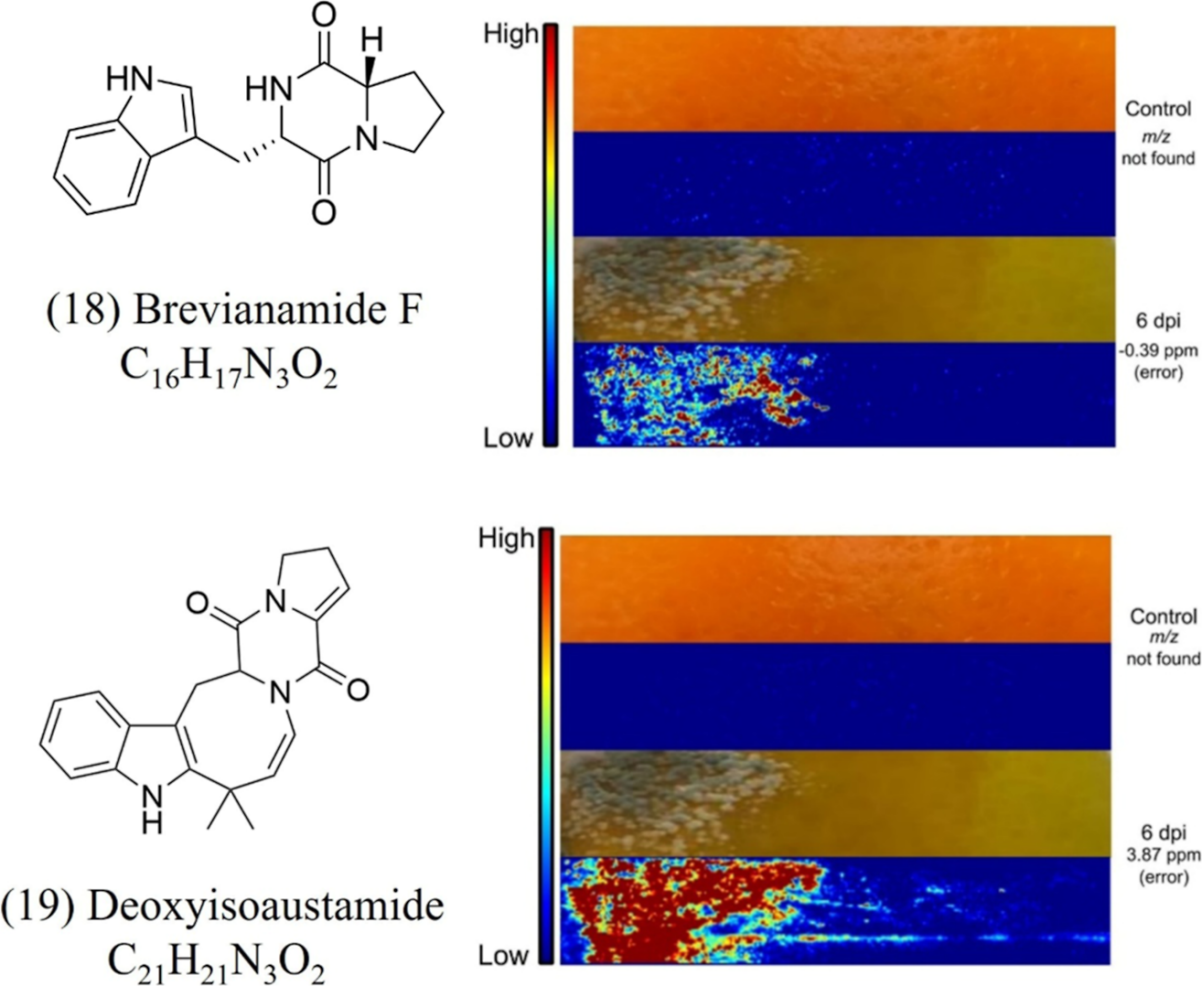
Spatial distribution of brevianamide F (18) and deoxyisoaustamide (19) in *P. italicum*-infected oranges using DESI-MSI. Ion images show metabolite localization in control and infected samples at 6 days post-inoculation. The respective *m*/*z* values were not detected in the control orange peels, while strong signals were observed in infected tissues, particularly at the fungal infection site. The color scale indicates relative ion intensity (from low in blue to high in red). Observed mass errors are −0.39 ppm for brevianamide F and 3.87 ppm for deoxyisoaustamide.

### 3.5 In Vitro Production of Diketopiperazine Alkaloids by*P. italicum*

In order to confirm the fungal origin of the six diketopiperazine derivatives detected in the pathogen-host interaction, *P. italicum* was cultivated in vitro in three different culture media. LC-HRMS analyses confirmed the presence of all ions at [M + H]^+^ *m*/*z* 282.1236; 284.1394; 348.1707; 350.1860; 352.2021; and 366.1808, corresponding to the six diketopiperazine alkaloids detected during infection (Figure S4). The MS/MS spectra of each ion were manually compared with the spectra obtained from in vivo analyses and cross-referenced with databases from the GNPS platform. These findings confirm that the metabolites 12,13-dehydroprolyltryptophanyldiketopiperazine, brevianamide F, deoxyisoaustamide, deoxybrevianamide E, dehydrodeoxybrevianamide E, and brevianamide A, previously identified during the progression of blue mold disease (Figure 2), are biosynthesized by*P. italicum* under in vitro conditions.

To confirm brevianamide F identification, a synthetic standard was chemically synthesized following a previously reported procedure^28^ (Supporting Information). The ^1^H and ^13^C NMR spectra confirmed the compound’s structure, showing chemical shifts (δ, ppm) consistent with previously reported values for brevianamide F.^37^ In addition, brevianamide F was purified from the in vitro fermentation of *P. italicum* to determine its absolute configuration. The production of this metabolite has never been directly related to *P. italicum*, being described for the first time as a natural product in this fungal species. Furthermore, the absolute configuration of brevianamide F produced by *P. italicum* was determined using Marfey’s reaction (Figures S5–S7), confirming the presence of l-proline and l-tryptophan as its stereochemical components.

### 3.6 Isolation and Identification of Endophytic Fungi Community from *Citrus sinensis*

Two microorganisms were isolated from healthy*C. sinensis* peel as endophytes and identified through sequencing and genetic distance analysis based on the ribosomal gene spacer region (ITS). The ITS region sequence (Figure S9) of one isolated microorganism exhibited 99–100% similarity to sequences from the same region of the ribosomal operon of *Colletotrichum* sp. A genetic distance analysis retrieved the sample within a low-resolution cluster (27%) with the strains*C. gloeosporioides* ICMP 17821 and*C. proteae* CBS 132882, suggesting the final identification as*Colletotrichum* sp., *gloeosporioides* complex (Figure S10).

For the other isolated microorganism, the ITS region sequence exhibited 98–99% similarity with sequences from the same region of the ribosomal operon of*Diaporthe* sp. that are deposited in the GenBank and Mycobank databases. The genetic distance analysis retrieved the sample within a low-resolution cluster (67%) with the strains*Diaporthe infecunda* CBS 133812, suggesting the final identification as *Diaporthe* sp., closely related to*D. infecunda* (Figure S10).

### 3.7 Coculture Assays between Endophytes and*P. italicum*

To assess the impact of *P. italicum* infection on the endophytic community of*C. sinensis*, in vitro coculture experiments between*P. italicum* and the two endophytes (*Colletotrichum* sp. and *Diaporthe* sp.) were performed. Data indicated a significant mutual inhibition between*P. italicum* and both endophytic fungi*Diaporthe* sp. and *Colletotrichum* sp. (Figure S8). *P. italicum* exhibited inhibitory effects on the mycelial growth of both *Diaporthe* sp. and*Colletotrichum* sp., reducing their growth rates by 16% and 21%, respectively, when compared to their monocultures (Figure S8).

Metabolic profile analysis by LC-HRMS applied in the confrontation zone of each coculture indicated that the five indole diketopiperazine alkaloids, including brevianamide F (Figure 4), were detected. These results suggest that these secondary metabolites could participate in antifungal defense mechanisms against fungal endophytes, providing competitive advantages during citrus host infection.

**Figure 4:**
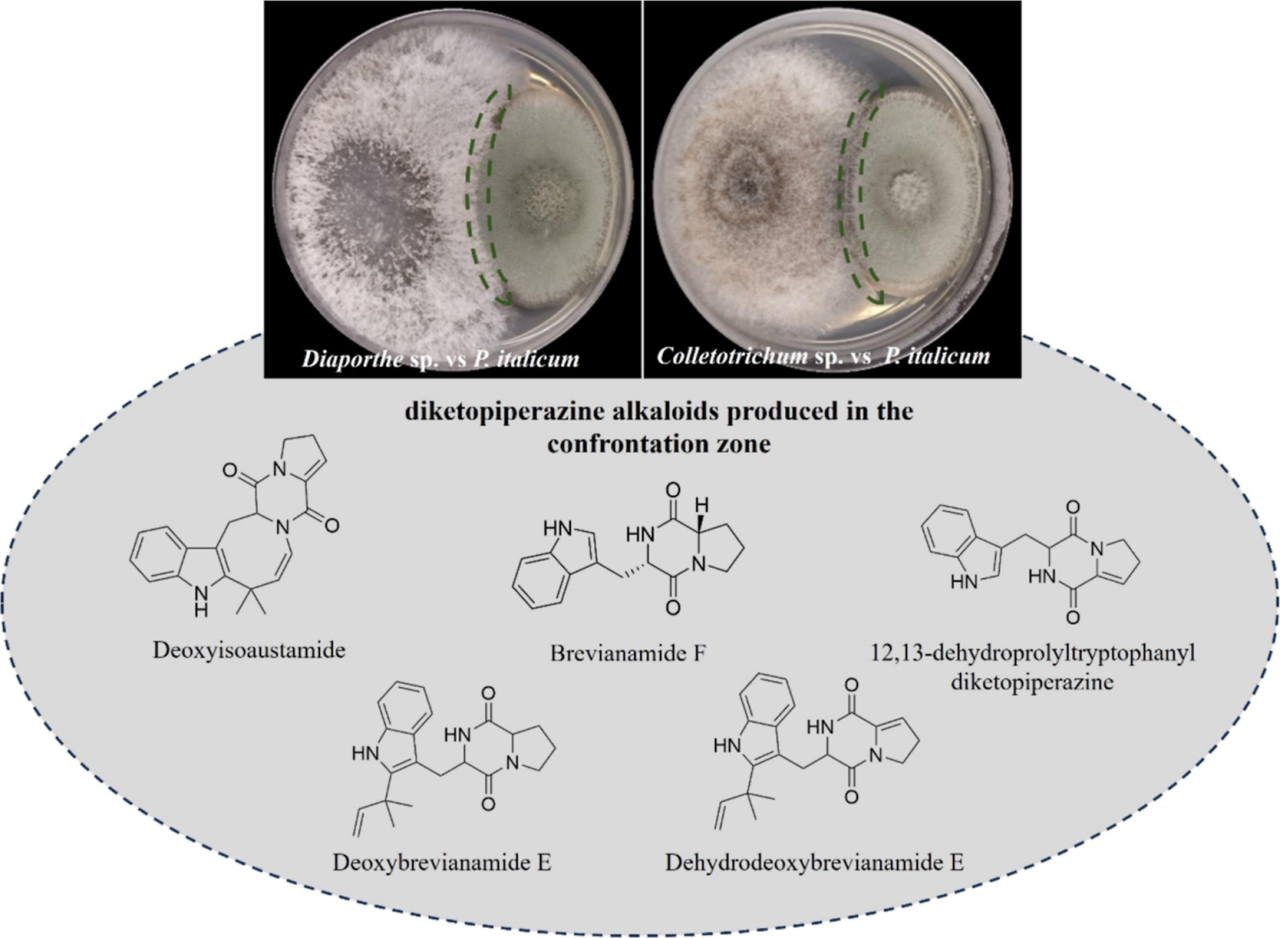
Confrontation assay between*P. italicum* and endophytic fungi. (Upper): coculture assays showing the interaction zones between*P. italicum* and*Diaporthe* sp. (left) or*P. italicum* and*Colletotrichum* sp. (right). Dashed green lines highlight the confrontation zone. (Lower): diketopiperazine alkaloids detected in the confrontation zone through LC–HRMS analysis and structure annotation: deoxyisoaustamide, brevianamide F, 12,13-dehydroprolyltryptophanyldiketopiperazine, deoxybrevianamide E, and dehydrodeoxybrevianamide E.

### 3.8 Brevianamide F Antifungal Assays with Citrus Endophytes

Brevianamide F, produced by*P. italicum*, was first evaluated at three concentrations (0.05, 0.10, and 0.30 mg/mL) against the endophytic fungi*Diaporthe* sp. and *Colletotrichum* sp. The compound inhibited the radial growth of*Diaporthe* sp. only at the highest concentration tested (0.30 mg/mL) (Figure 5), whereas no inhibitory effect was observed against*Colletotrichum* sp. at any concentration (data not shown). Based on this observation, a second assay was performed exclusively with*Diaporthe* sp. to further characterize the antifungal activity of brevianamide F. Growth inhibition was dose-dependent, ranging from 19.1% at 0.15 mg/mL, 23.0% at 0.2 mg/mL, and 43.3% at 0.250 mg/mL (Figure S11). These results indicate that Brevianamide F exhibits moderate inhibitory activity against*Diaporthe* sp., with growth inhibition ranging from 19.1% at 0.15 mg/mL to 51.9% at 0.3 mg/mL.

**Figure 5:**
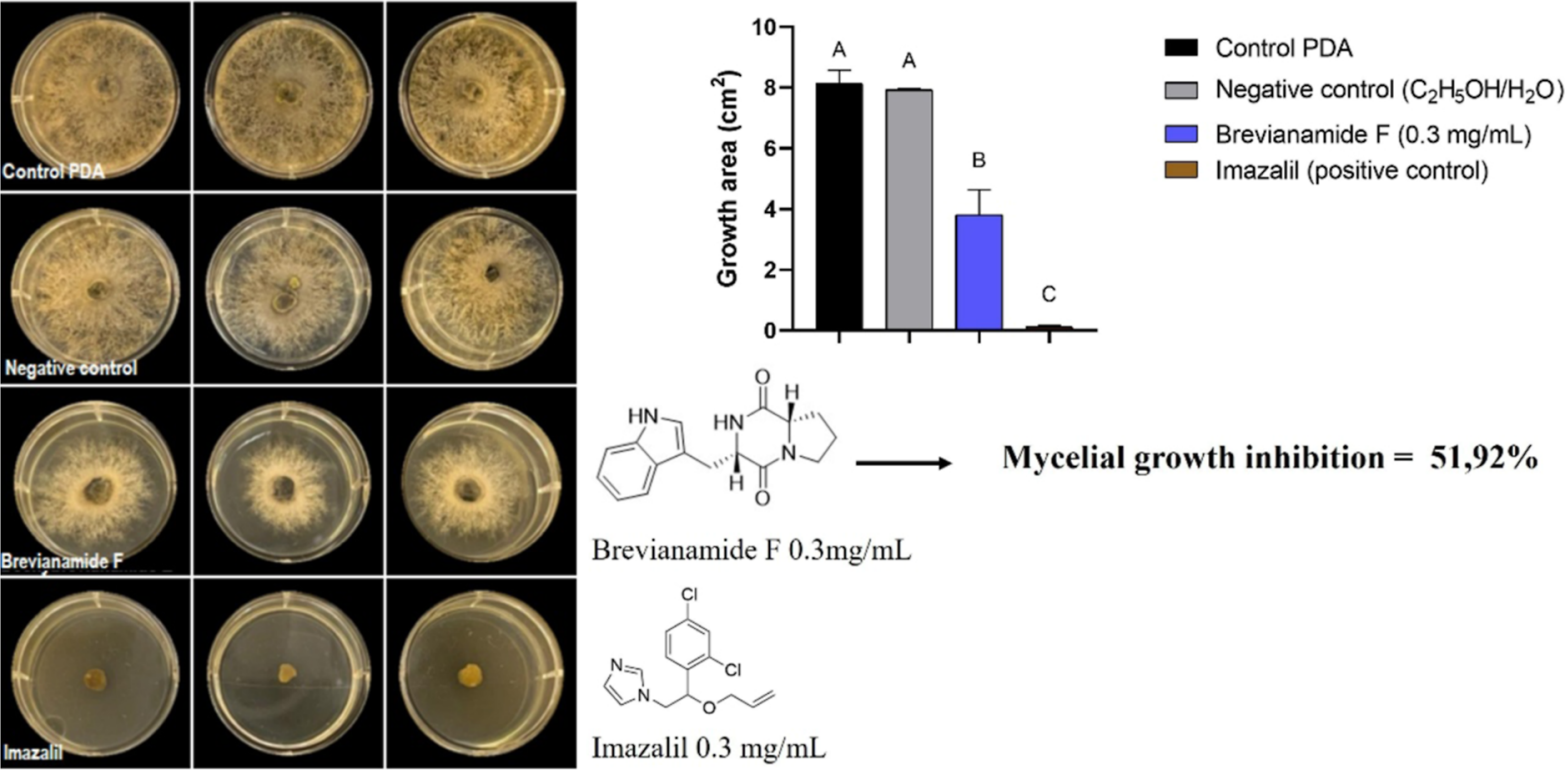
Antifungal activity of secondary metabolites against*Diaporthe* sp. (Left): mycelial growth of *Diaporthe* sp. on PDA plates after 7 days of incubation under four treatments: control (PDA only), negative control (ethanol/water 1:1, v/v), brevianamide F (0.3 mg/mL), and imazalil (0.3 mg/mL, positive control). (Right, upper): quantification of fungal growth area (cm^2^). Data represent mean ± standard deviation (*n* = 3). Statistical analysis was performed using one-way ANOVA (*p* < 0.0001), followed by Tukey’s post hoc test. Treatments labeled with the same letter are not significantly different, while those with different letters indicate statistically significant differences. (Right, lower): chemical structures of brevianamide F and imazalil used in the treatments. Brevianamide F inhibited mycelial growth by 51.92% compared to the control.

### 3.9 Confocal Microscopy Reveals the Inhibitory Effect of Brevianamide F on *Diaporthe* sp. Hyphal Development

Confocal laser scanning microscopy analysis of*Diaporthe* sp. hyphae treated with brevianamide F revealed an irregular staining pattern (Figure 6). In contrast, the untreated*Diaporthe* sp. hyphae (control) displayed uniform Congo red staining throughout the fungal region (Figure 6). Congo red binds specifically to β-1,4 polysaccharides such as chitin, which is a major component of the fungal cell wall. These observations suggest that brevianamide F may disrupt the integrity of the*Diaporthe* sp. cell wall.^38^

**Figure 6:**
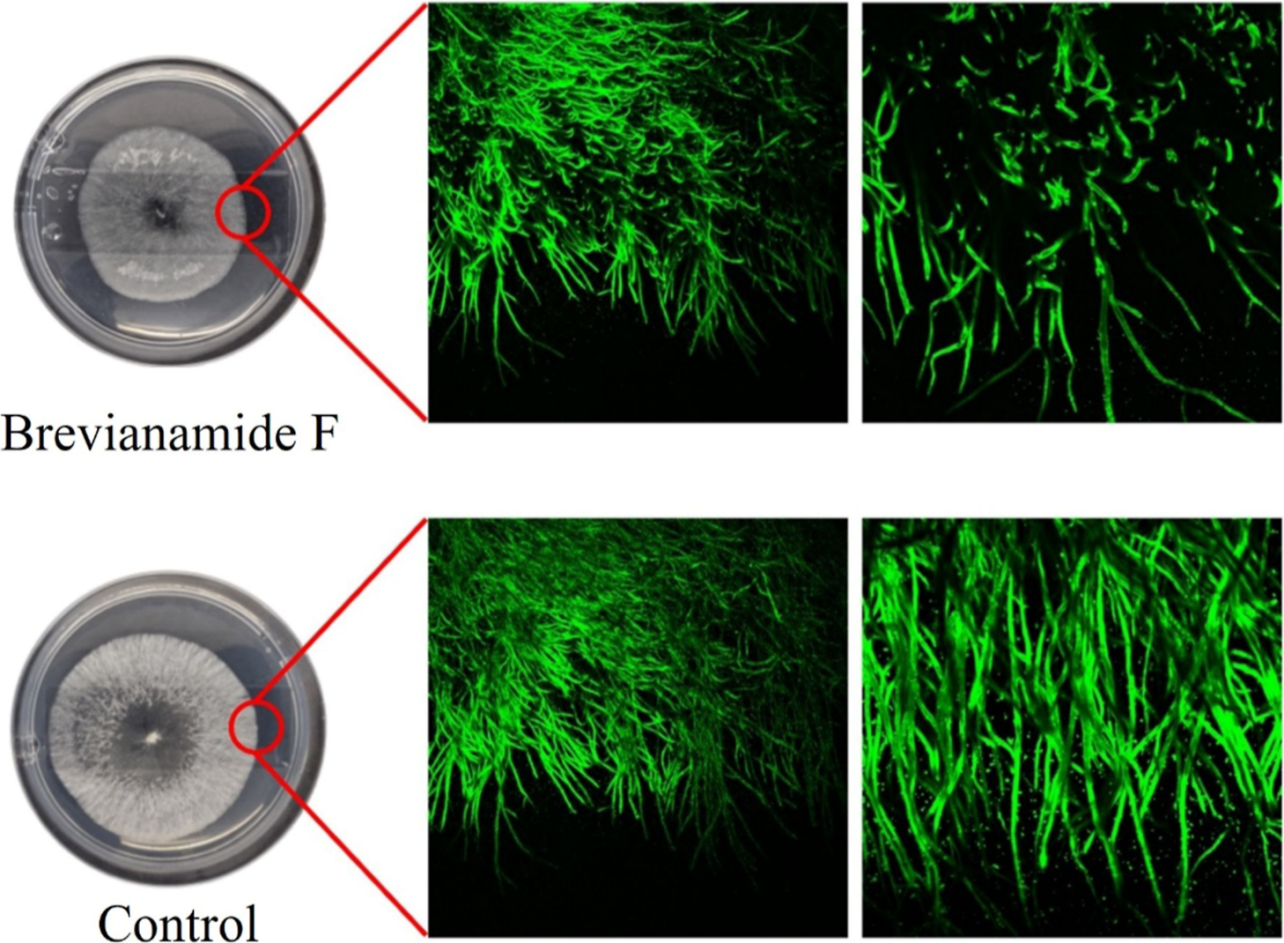
Effect of brevianamide F on fungal hyphal morphology. Representative images of*Diaporthe* sp. grown on PDA plates supplemented with brevianamide F (upper row) or control PDA (lower row). Left panels show colony morphology, while middle and right panels display confocal microscopy images of the hyphal structure stained with a fluorescent dye. Treatment with brevianamide F results in notable alterations in hyphal organization and density compared to the untreated control.

## 4 Discussion

Metabolomics analysis plays a fundamental role in understanding the biochemical responses of plants to microbial infection.^24^ The integration of LC-HRMS and DESI-MSI techniques in this study provided an integrated approach, combining the detailed analysis of metabolites with their spatial visualization. MSI has been previously used to analyze the surfaces of orange peels infected by*P. digitatum*, successfully detecting tryptoquialanines A and B and other intermediates in the tryptoquialanine biosynthetic pathway.^39^ In this study, we report the novel application of MSI for monitoring metabolites produced during the interaction of *P. italicum* with the*Citrus sinensis*host.

Multivariate data analysis provided strong evidences of metabolic changes in*P. italicum*-infected oranges compared to non-infected controls. The PCA results showed a clear separation between the two groups, indicating that the infection induces significant metabolic alterations, distinguishing between infected and healthy control samples. This clustering pattern reflects a coordinated metabolic response of the fruit to pathogen colonization, underscoring a shift in the biochemical composition. The identification of specific metabolic features driving this differentiation provides valuable insights into the fruit–pathogen interaction.

The use of the GNPS library, in conjunction with other natural products databases, enabled the annotation of 22 metabolites produced during *P. italicum*–*citrus* interaction, including phenylpropanoids, which play a pivotal role in citrus defense mechanisms.^40^

Hesperetin 7-*O*-glucoside (Table 1, compound 14) is a flavanone glycoside naturally abundant in *Citrus sinensis* fruits,^41^ where it plays a key role in the plant’s antioxidant system and contributes to defense responses against biotic stresses.^42,43^ In our study, the significant increase in hesperetin 7-*O*-glucoside levels during blue mold infection suggests that the host plant responds to*P. italicum* colonization by enhancing the biosynthesis or accumulation of this flavonoid derivative. This response could be associated with the activation of the phenylpropanoid pathway, commonly involved in plant defense mechanisms.^44^ Flavanone glycosides like hesperetin-7-*O*-glucoside have been reported to exhibit antimicrobial and antifungal properties, potentially limiting pathogen growth or signaling further immune responses.^45^ Similar observations have been made in other plant-pathogen systems, where the accumulation of flavonoid glycosides is part of an induced defense mechanism.^46^ Therefore, the increased presence of hesperetin 7-*O*-glucoside during infection may represent a metabolic marker of the host’s response and could contribute to understanding the chemical landscape of citrus resistance or susceptibility to*P. italicum*.

Diosmin (Table 1, compound 15) is a glycosylated flavone widely distributed in citrus species, known for its strong antioxidant and anti-inflammatory properties.^42,43^ In the present study, the concentration of diosmin significantly increased in orange fruits infected with*P. italicum*, suggesting that this metabolite may be involved in the host’s defense response to infection. Previous studies have shown that flavones like diosmin can alleviate oxidative stress triggered by pathogens and modulate signaling pathways related to plant defense.^47,48^ Therefore, its accumulation may reflect an adaptive response of the plant to limit fungal progression and mitigate tissue damage. These findings highlight the possibility that*C. sinensis* modulates polymethoxyflavone production as part of its metabolic adaptation to pathogen invasion. Further studies are needed to elucidate the specific roles of these compounds in plant defense and their potential impact on the blue mold infection dynamics.

Feruloyl putrescine (Table 1, compound 7) is another metabolite produced by*C. sinensis*. Synthesized through two biosynthetic pathways, it plays a role in the fruit’s response to *Candidatus Liberibacter asiaticus*.^49^ This compound exhibits diverse biological activities, including antioxidant, anticancer, and antimicrobial activities.^49^ Some studies highlight its antimicrobial activity against pathogenic bacteria such as *Staphylococcus aureus* and *Escherichia coli*. Furthermore, feruloyl putrescine demonstrates antifungal properties against*Aspergillus niger*, while its derivative, feruloyl putrescine hydrochloride, exhibits antifungal activity against*Penicillium verrucosum*.^50^

Another compound previously reported in citrus,^51^ 3′,5,7-trihydroxyflavanone (Table 1, compound 8), also annotated in this study, demonstrates antifungal properties against *Penicillium notatum*, with a MIC value of 0.8 mg·mL^–1^.^52^ Furthermore, Yang et al. propose 3′,5,7-trihydroxyflavanone, a major flavonoid in Chinese propolis, as a potential natural alternative for controlling*Citrus* blue mold caused by*P. italicum*.^53^

The six diketopiperazine alkaloids identified in the interaction between*P. italicum* and citrus are described as microbial secondary metabolites that have been previously produced by*Penicillium* spp.^54,55^ These alkaloids are noted for their diverse pharmacological activities.^56^ Brevianamide F (Table 1, compound 18) is produced by several fungal species, including*Penicillium brevicompactum*,^57^ *Penicillium vinaceum*,^58^ and*Aspergillus fumigatus*.^59,60^ Recent studies have identified brevianamide F as a precursor of 12,13-dehydrodesoxybrevianamide E, a metabolite previously found in the in vitro cultures of*P. italicum*.^61^ Furthermore, Asiri et al. described the antimicrobial activity of brevianamide F against *S. aureus* and *Candida albicans*.^58^ However, there is no evidence regarding the biological role of either brevianamide F or 12,13-dehydrodesoxybrevianamide E in the context of blue mold disease.

Deoxybrevianamide E (Table 1, compound 21) was previously isolated from the fungal mycelia of*P. italicum* and has been identified as an extracellular metabolite of this species.^7,62^ Its production has also been observed in other fungal species, such as *Aspergillus ustus*,*Aspergillus protuberus*, and*Penicillium ulaiense*,^62–64^ and it is the precursor of brevianamides A, B, and E.^65^ Deoxybrevianamide E is a member of the brevianamide family of prenylated indole alkaloids. While several brevianamides have demonstrated various biological activities, such as antibacterial, anti-insect, and antituberculosis properties, currently, there is no information available on the bioactivity of deoxybrevianamide E.^66^

Deoxyisoaustamide (Table 1, compound 19) belongs to the deoxyaustamides class of alkaloids, characterized by the azocino­[5,4-*b*] indole regioisomer.^67^ Production of this alkaloid has been reported in various*Penicillium* species,^68,69^ but not yet during the interaction between*P. italicum* and citrus. Although the biological role of deoxyisoaustamide in citrus infection remains unknown, its derivatives have shown cytotoxic and neuroprotective activities induced by glutamate and *tert*-butyl hydroperoxide.^69^

Given the similarities of diketopiperazine alkaloids with other*Penicillium*-produced metabolites and their reported antifungal biological activities, we hypothesized that the identified indole diketopiperazine alkaloids have similar antimicrobial effects. This hypothesis was tested through coculture experiments between *P. italicum* and the endophytic microorganisms isolated from healthy*C. sinensis* peels (*Diaporthe* sp. and *Colletotrichum* sp.). Our data confirmed the presence of the same diketopiperazine alkaloids in the interface zone between endophytes and the pathogen, suggesting a potential antimicrobial strategy against the endophytic community during infection.

Brevianamide F demonstrated antimicrobial activity against*Diaporthe* sp., with the fungus exhibiting an irregular hypha pattern upon exposure to this compound. This observation is consistent with previous studies by Costa et al., where*P. digitatum* showed defective hyphae upon exposure to secondary metabolites produced during its interaction with*Penicillium citrinum*.^35^ Therefore, the confocal microscopy analysis and antifungal assays conducted with brevianamide F further support its antifungal potential against the endophyte *Diaporthe* sp., highlighting its impact on cellular morphology, particularly in causing characteristic defects in the fungal cell wall. This may indicate that brevianamide F could be produced as a component in the offensive strategy of *P. italicum* against endophytic microorganisms during the establishment of blue mold disease.

In contrast, no inhibitory effect was observed against*Colletotrichum* sp. Notably, our LC-HRMS data revealed that the strain isolated from orange peel also produces brevianamide F. This endogenous biosynthesis likely confers an intrinsic resistance, as fungi frequently evolve self-protection mechanisms, such as efflux pumps, target site modifications, or detoxification enzymes, to neutralize secondary metabolites they produce.^70^ Additionally, the constitutive presence of brevianamide F within *Colletotrichum* cells may lead to adaptive changes in membrane composition or cell wall structure, further reducing the susceptibility. This self-resistance phenomenon has been widely reported in microbial secondary metabolism and may explain the differential sensitivity observed between the two endophytic fungi tested.^71^

This study provides a comprehensive view of the metabolic dynamics in the*C. sinensis*–*P. italicum* pathosystem. LC-HRMS analyses revealed distinct metabolic profiles between healthy and infected fruits, identifying key metabolites linked to citrus defense responses, including phenylpropanoids, and offering a broader understanding of pathogen–host metabolic interactions. Six indole diketopiperazine alkaloids were detected in infected tissues for the first time, and MSI confirmed their exclusive localization in the infected peels. Coculture assays with *C. sinensis* endophytes and *P. italicum* demonstrated mutual inhibition, with indole diketopiperazine alkaloids, among them brevianamide F, present in the interaction zone. Antifungal assays indicated that brevianamide F inhibits *Diaporthe* sp., suggesting a role in *P. italicum*’s strategy to compete with endophytic fungi during the disease establishment. The results connect the secondary metabolism of *P. italicum* to the infection process and provide valuable insights that could enable future strategies for controlling this disease.

## Supplementary Material



## Data Availability

All data generated or analyzed during this study are included in this published article and its Supporting Information.

## Notes

Synthesis and NMR analysis of brevianamide F; LC–MS TICs of orange peel extracts; PCA and PLS-DA plots of metabolomic data; MS/MS molecular network showing metabolite differences between healthy and infected samples; in vitro LC–MS/MS confirmation of diketopiperazines; FDAA derivatization HPLC-UV chromatograms for proline stereochemistry; FDAA derivatization HPLC-UV chromatograms for tryptophan stereochemistry; EICs for d- and l-tryptophan FDAA derivatives and brevianamide F; dual-culture assays with endophytic fungi; ITS sequences of isolates; phylogenetic dendrograms of *Colletotrichum* sp. and *Diaporthe* sp. isolates; and MIC assay of brevianamide F against *Diaporthe* sp. (PDF)
