# Supplementary material for: Decoding the Penicillium italicum–Citrus Interaction: Untargeted Metabolomics Sheds Light on a Neglected Postharvest Pathogen
Source: J Agric Food Chem. 2025 Oct 16;73(43):27806–19. doi: 10.1021/acs.jafc.5c07618 (PMC12576830; doi:10.1021/acs.jafc.5c07618)
Supplement: Supplementary file 1 [file jf5c07618_si_001.pdf]

Supporting Information:

Decoding the *Penicillium italicum–Citrus* Interaction: Untargeted Metabolomics Sheds Light on a Neglected Postharvest Pathogen

Evandro Silva[a,b#,], Aline Midori Kanashiro[a#], José Rodrigo Ferreira Maciel[a], Rodolfo Dantas Lima Junior[a], Maria Antonia Fraga Botelho[a], Alana Kelyene Pereira[a], Stephanie Nemesio da Silva[a], Jonas Henrique Costa[a], João Guilherme de Moraes Pontes[a], Amanda Ferreira da Silva[a], Igor Dias Jurberg[a], Roberto G. S. Berlinck[b,*], Taicia Pacheco Fill[a,*]

[a]Universidade Estadual de Campinas, Instituto de Química, CEP, 13083-970 Campinas, SP, Brazil.

[b]Instituto de Química de São Carlos, Universidade de São Paulo, CP 780, CEP 13560-970, São Carlos, SP, Brazil.

#These authors contributed equality.

Corresponding authors: rgsberlinck@iqsc.usp.br and taicia@unicamp.br

Synthesis and NMR Analysis of Brevianamide F

(3S,8aS)-3-((1H-indol-3-yl)methyl)hexahydropyrrolo[1,2-a]pyrazine-1,4-dione (Brevianamide F)

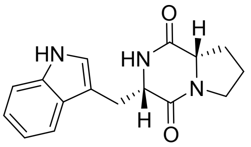

This compound was prepared as previously reported in the literature.[1,2]

^{1}H (CDCl$_3$, 250 MHz) δ: 8.47 (br s, 1H), 7.59 (d, *J* = 8.0 Hz, 1H), 7.39 (d, *J* = 8.0 Hz, 1H), 7.23 (td, *J* = 8.0 Hz, *J* = 1.3 Hz, 1H), 7.13 (td, *J* = 8.0 Hz, *J* = 1.3 Hz, 1H), 7.07 (d, *J* = 2.5 Hz, 1H), 5.80 (br s, 1H), 4.37 (dd, *J* = 10.5 Hz, *J* = 2.5 Hz, 1H), 4.10 – 4.03 (m, 1H), 3.75 (dd, *J* = 15.0 Hz, *J* = 3.6 Hz, 1H), 3.69 – 3.53 (m, 2H), 2.98 (dd, *J* = 15.0 Hz, *J* = 10.5 Hz, 1H), 2.38 – 2.27 (m, 1H), 2.10 – 1.86 (m, 3H).

^{13}C{^{1}H} (CDCl$_3$, 62.5 MHz) δ: 169.5, 165.7, 136.8, 126.8, 123.5, 122.8, 120.0, 118.6, 111.7, 109.9, 59.3, 54.7, 45.5, 28.4, 27.0, 22.7.

[1] For the preparation of Brevianamide F, see: Kieffer, M. E.; Chuang, K. V.; Reisman, S. E.; Copper-Catalyzed Diastereoselective Arylation of Tryptophan Derivatives: Total Synthesis of (+)-Naseseazines A and B. *J. Am. Chem. Soc.* **2013**, *135*, 5557–5560. DOI:10.1021/ja4023557

[2] ^{1}H and ^{13}C{^{1}H} NMR spectra are in good agreement with the literature. See: Caballero, E.; Avendaño, C.; Menéndez, J. C.; Brief Total Synthesis of the Cell Cycle Inhibitor Tryprostatin B and Related Preparation of its Alanine Analogue. *J. Org. Chem.* **2003**, *68*, 6944–6951. DOI:10.1021/jo034703l.

Brevianamide F: ^{1}H NMR ($CDCl_3$, 250 MHz)

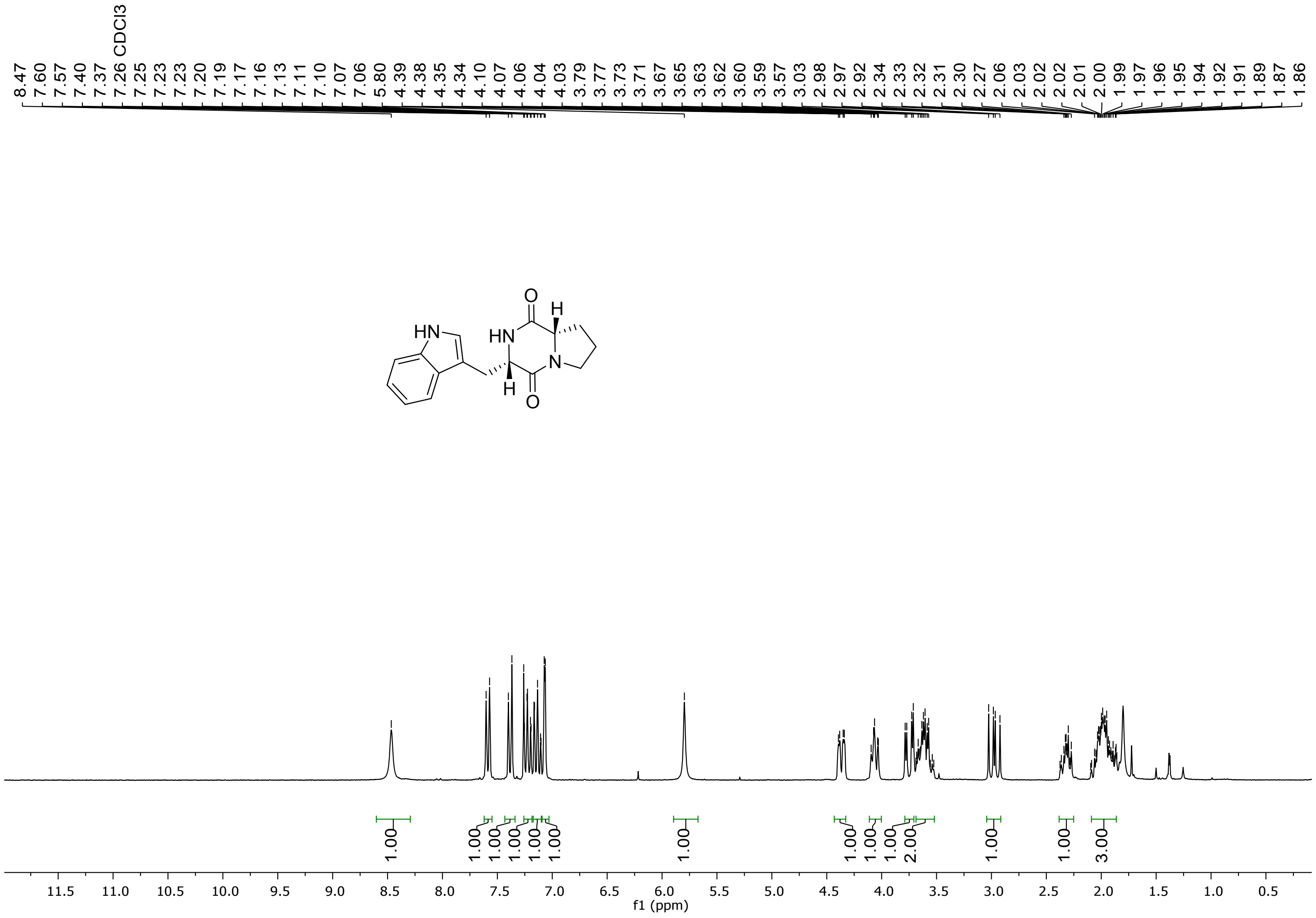

Brevianamide F: $^{13}C\{^{1}H\}$ NMR ($CDCl_3$, 62.5 MHz)

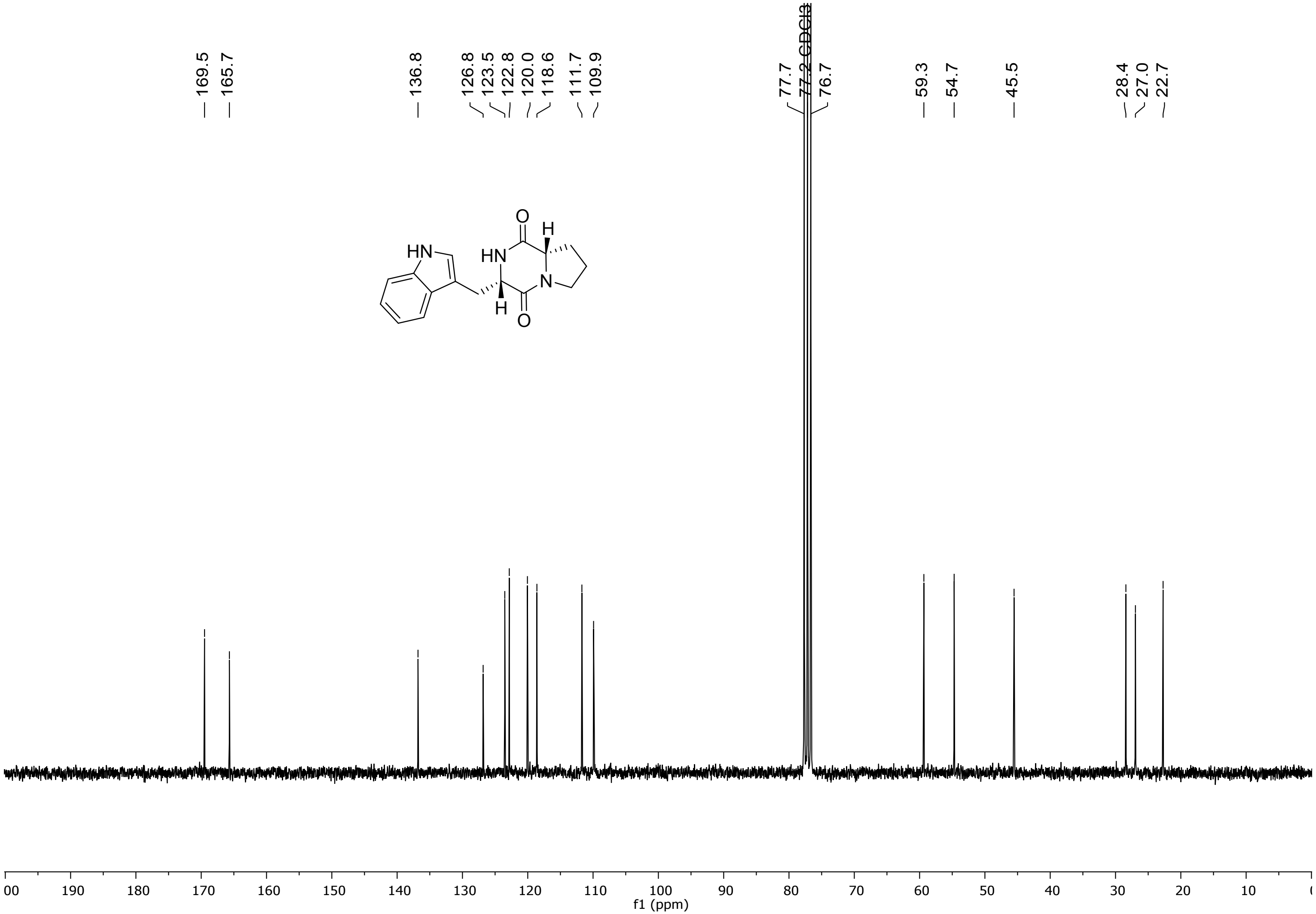

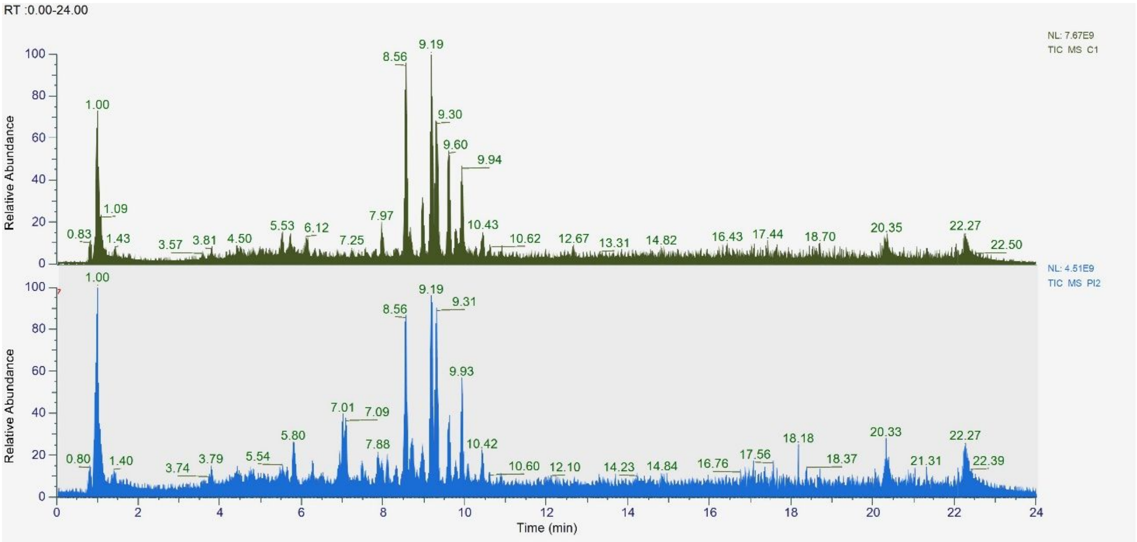

Figure S1. Total ion chromatograms (TIC) obtained from untargeted LC-HRMS analysis (positive ion mode) of orange peel extracts. The upper panel (green) corresponds to non-inoculated control samples, and the lower panel (blue) represents samples inoculated with *P. italicum*. Differences in peak profiles indicate distinct metabolite production associated with fungal infection.

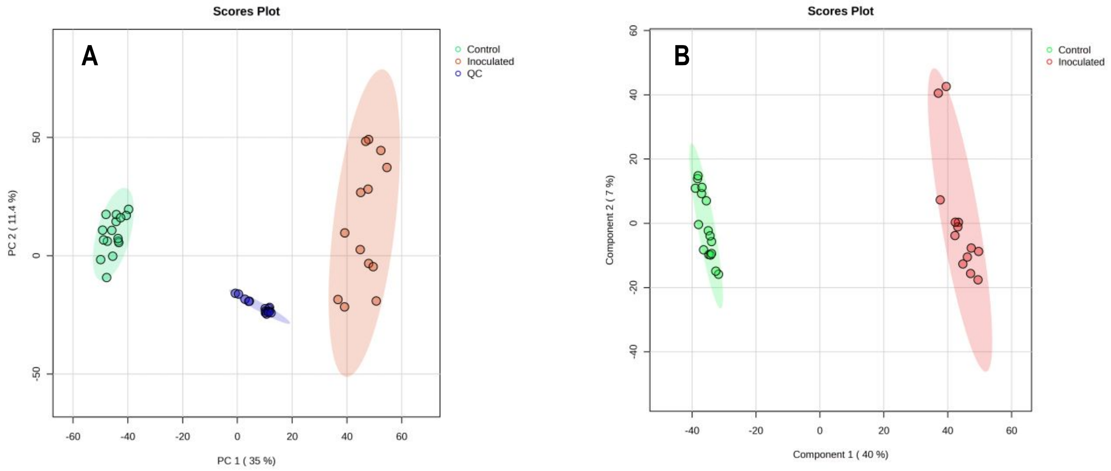

Figure S2. (A) PCA scores plot corresponding to Figure 1, here including QC samples (purple) to assess data quality. The QC replicates clustered tightly together and were clearly separated from the experimental samples, confirming the reliability of the dataset. The first five principal components explained 68.6% of the total variance. (B) PLS-DA scores plot showing distinct separation between control (green) and *P. italicum*-inoculated (red) citrus samples. Clustering along Component 1 (40%) and Component 2 (7%) indicates significant metabolic differences associated with infection. Each point represents an individual sample, and ellipses denote the 95% confidence interval for each group. Note: PC1 and PC2 refer to principal components in PCA (panel A), whereas Component 1 and Component 2 refer to the latent variables in PLS-DA (panel B).

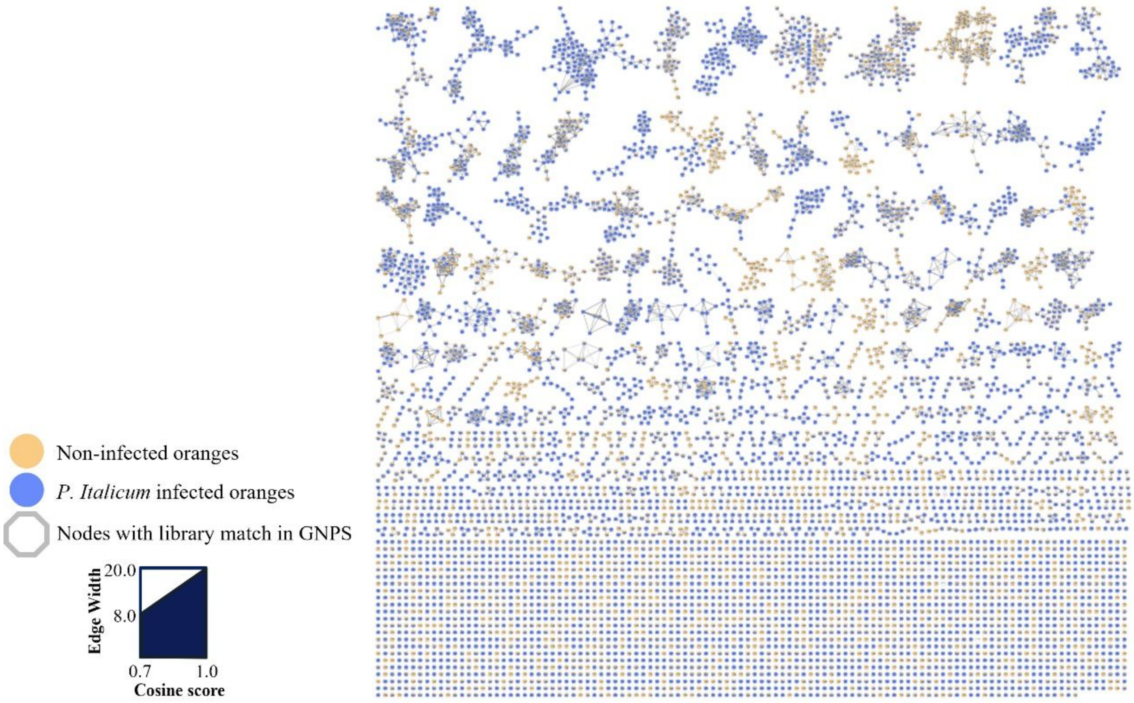

Figure S3. Molecular network generated from LC-HRMS data showing the distribution of metabolites detected in healthy orange samples (orange nodes) and *P. italicum* strain samples (blue nodes). Each node represents a unique molecular feature, and edges indicate spectral similarity based on cosine scores (edge thickness increases with higher similarity). Grey-outlined nodes represent metabolites that matched to known compounds in the GNPS spectral library. This network reveals distinct metabolic profiles between healthy and infected samples, highlighting candidate metabolites involved in the *P. italicum*–citrus interaction.

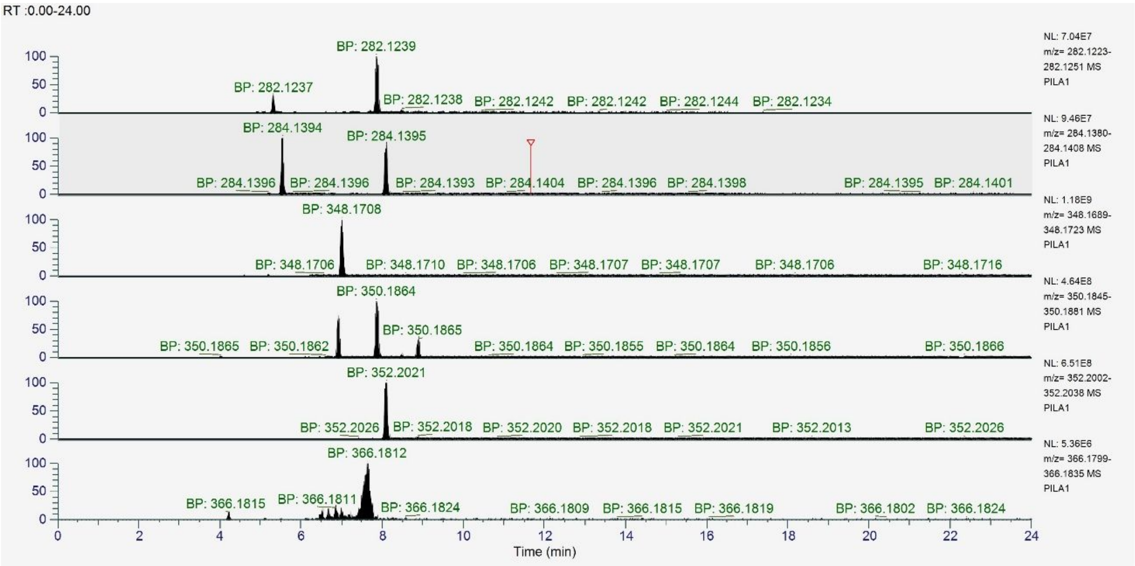

Figure S4. Extracted ion chromatograms (EICs) from in vitro LC-HRMS analysis of *P. italicum* culture extracts. The presence of the ions at $[M+H]^+$ *m/z* 282.1236; 284.1394; 348.1707; 350.1860; 352.2021; and 366.1808 confirms the production of the metabolites 12,13-dehydroprolyltryptophanyldiketopiperazine, brevianamide F, deoxyisoaustamide, deoxybrevianamide E, dehydrodeoxybrevianamide E, and brevianamide A, respectively. Compound identification was supported by MS/MS fragmentation data.

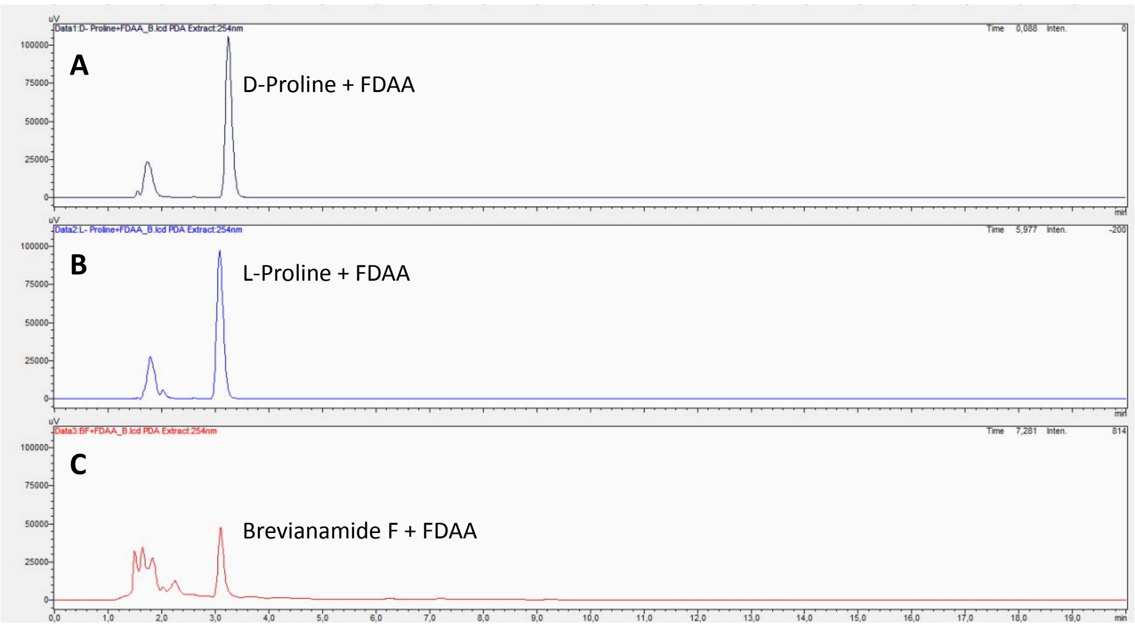

Figure S5. Derivatization with FDAA (1-fluoro-2,4-dinitrophenyl-5-L-alanine amide) for determination of proline stereochemistry by HPLC-UV at 254 nm. (A) Chromatogram of D-proline + FDAA showing retention time ~5.98 min; (B) chromatogram of L-proline + FDAA showing a similar retention time; (C) chromatogram of derivatized brevianamide F + FDAA, with a distinct peak at ~7.28 min, confirming the presence and stereochemistry of proline residue in the compound.

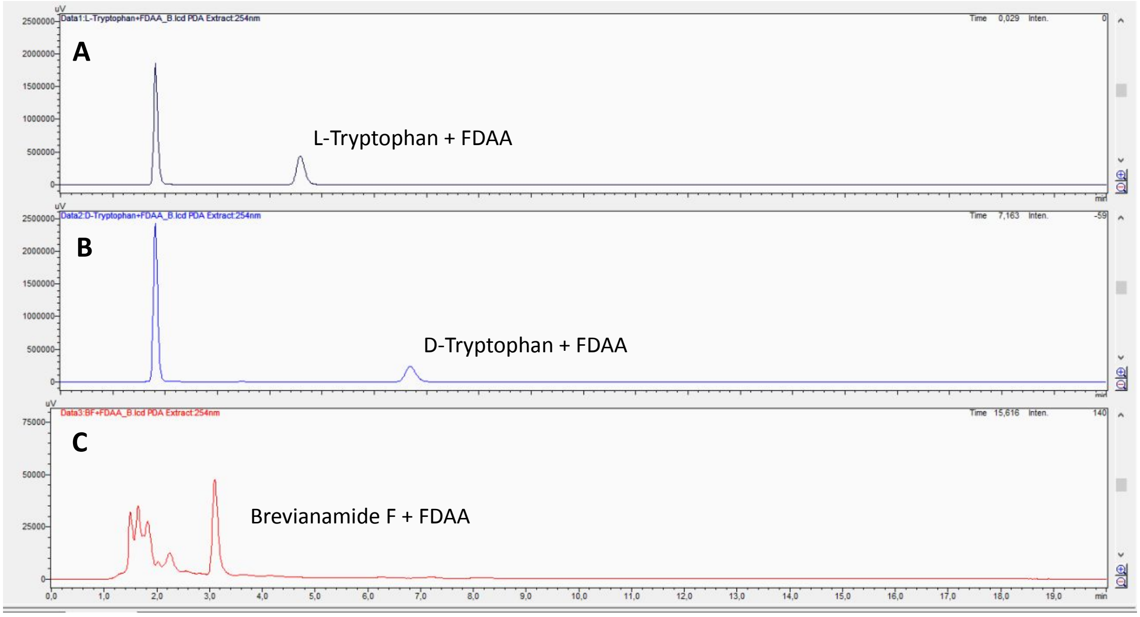

Figure S6. HPLC-UV chromatograms at 254 nm showing derivatization with FDAA (1-fluoro-2,4-dinitrophenyl-5-L-alanine amide) for stereochemical determination of tryptophan. (A) L-Tryptophan + FDAA shows a major peak at ~9.23 min; (B) D-Tryptophan + FDAA with a peak at ~7.85 min; (C) Brevianamide F + FDAA shows a retention time matching that of D-tryptophan, indicating the presence of a D-tryptophan residue in the molecule.

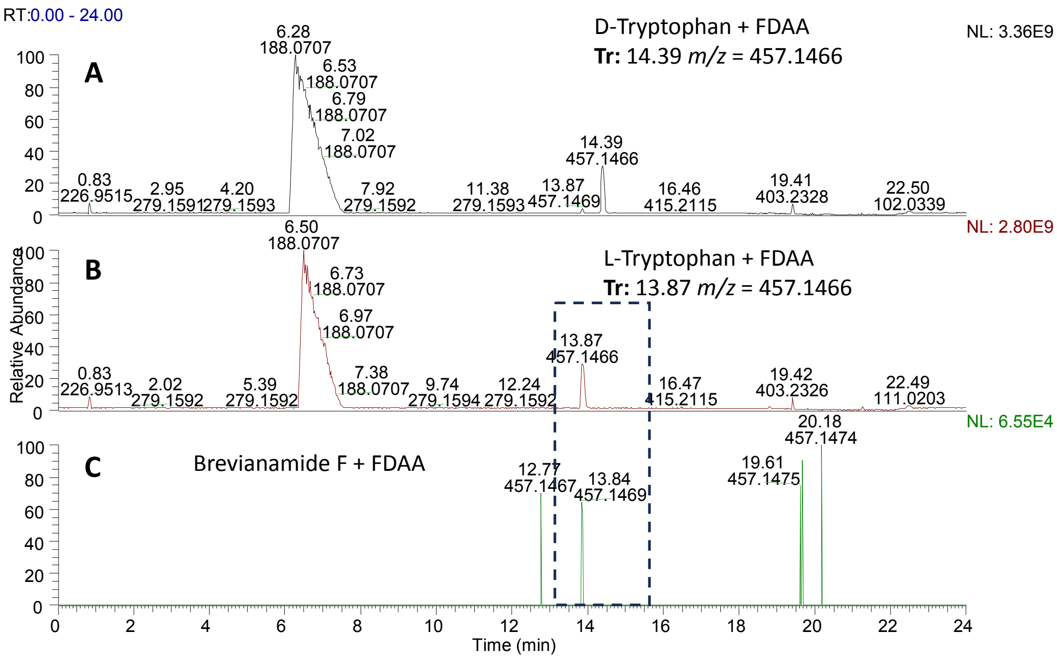

Figure S7. A) Extracted Ion Chromatogram 457.1466 D-Tryptophan + FDAA . (B) Extracted Ion Chromatogram 457.1466 L-Tryptophan + FDAA (C) Extracted Ion Chromatogram 457.1466 Brevianamide F + FDAA.

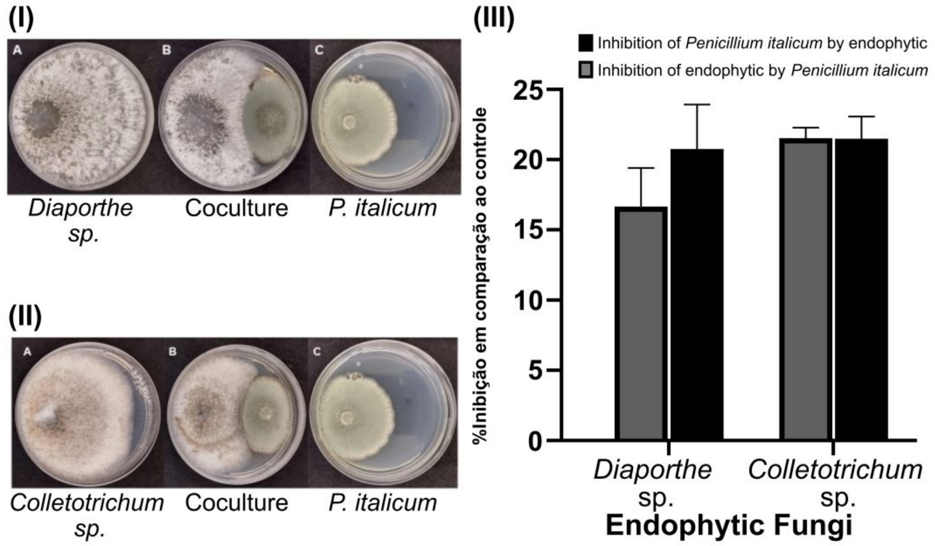

Figure S8. Dual-culture assay evaluating antagonistic interactions between *P. italicum* and endophytic fungi. (I) Representative images of the interaction between *Diaporthe* sp. and *P. italicum* grown alone or in coculture on PDA medium: (A) *Diaporthe* sp., (B) coculture, (C) *P. italicum*. (II) Representative images of the interaction between *Colletotrichum* sp. and *P. italicum*: (A) *Colletotrichum* sp., (B) *coculture*, (C) *P. italicum*. (III) Quantification of inhibition percentages of fungal growth in coculture compared to individual controls. Black bars represent inhibition of *P. italicum* by the endophyte, and gray bars represent inhibition of the endophyte by *P. italicum*. Values are expressed as mean ± standard deviation.

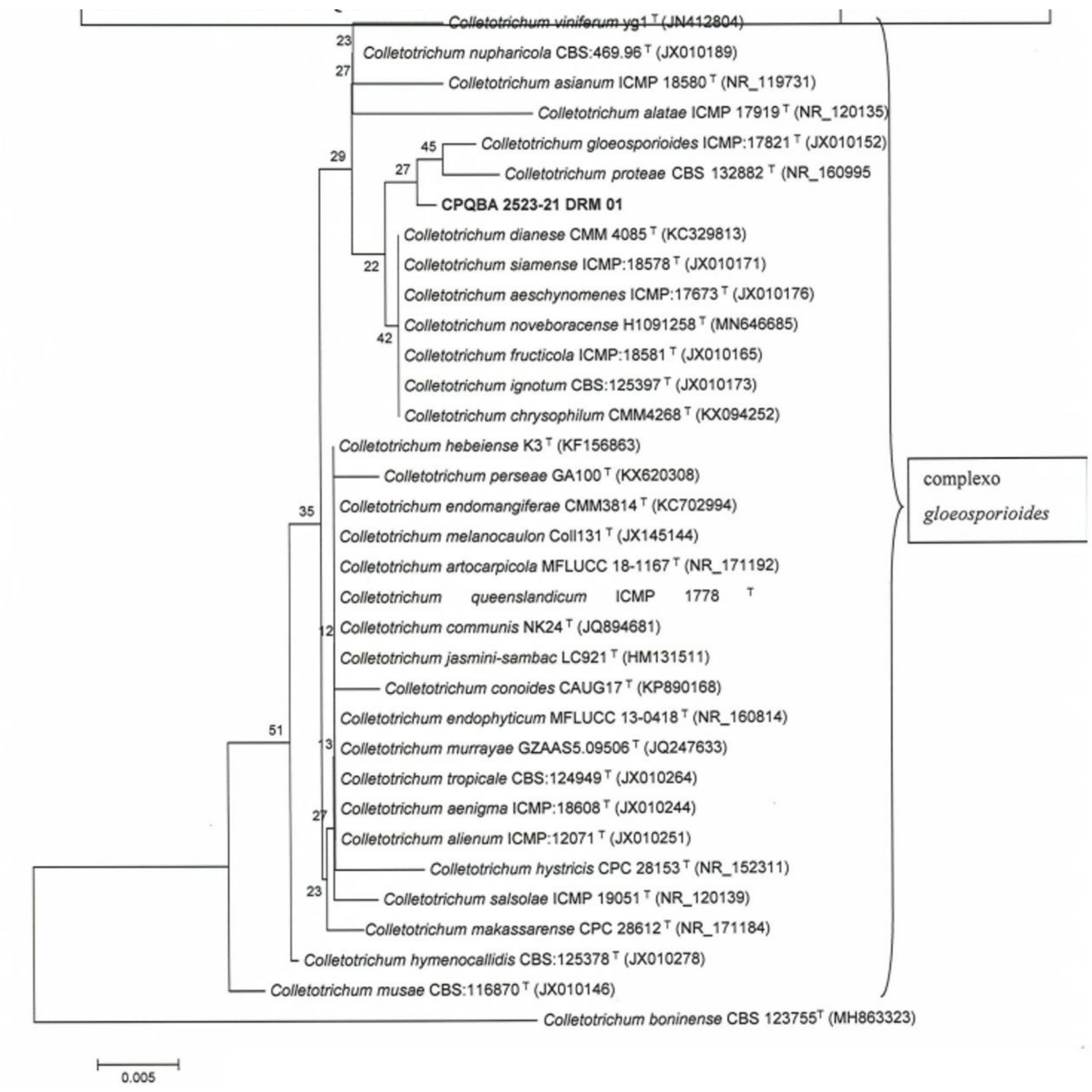

Figure S9. Dendrogram based on genetic distance, constructed using the *Neighbor-joining* method, showing the relationship between the partial ITS region sequence of the sample CPQBA 2532/21 DRM 01 and sequences of related microorganism strains available in the Mycobank database (CBS Knaw, currently Westerdijk Fungal Biodiversity Institute) and GenBank. Phylogenetic analysis places the sample within the *Colletotrichum gloeosporioides* complex, indicating its identification as belonging to the genus *Colletotrichum*.

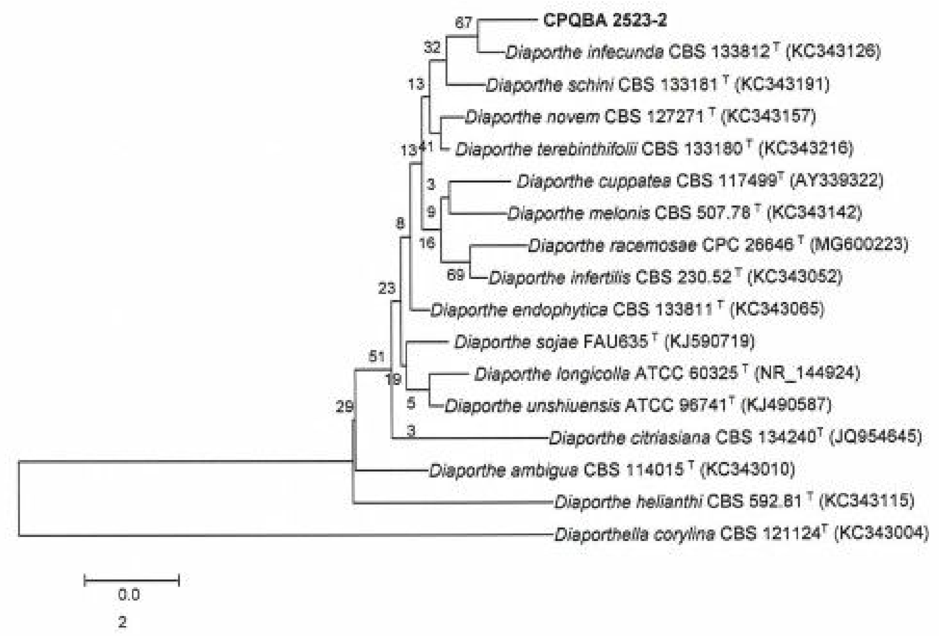

Figure S10. Dendrogram based on genetic distance, constructed using the *Neighbor-joining* method, showing the relationship between the partial ITS region sequence of the sample CPQBA 2523/21 DRM 02 and sequences of related microorganism strains available in the Mycobank database (CBS Knaw, currently Westerdijk Fungal Biodiversity Institute) and GenBank. Phylogenetic analysis places the sample in the same clade as *Diaporthe infecunda*, indicating its identification as belonging to the genus *Diaporthe*.

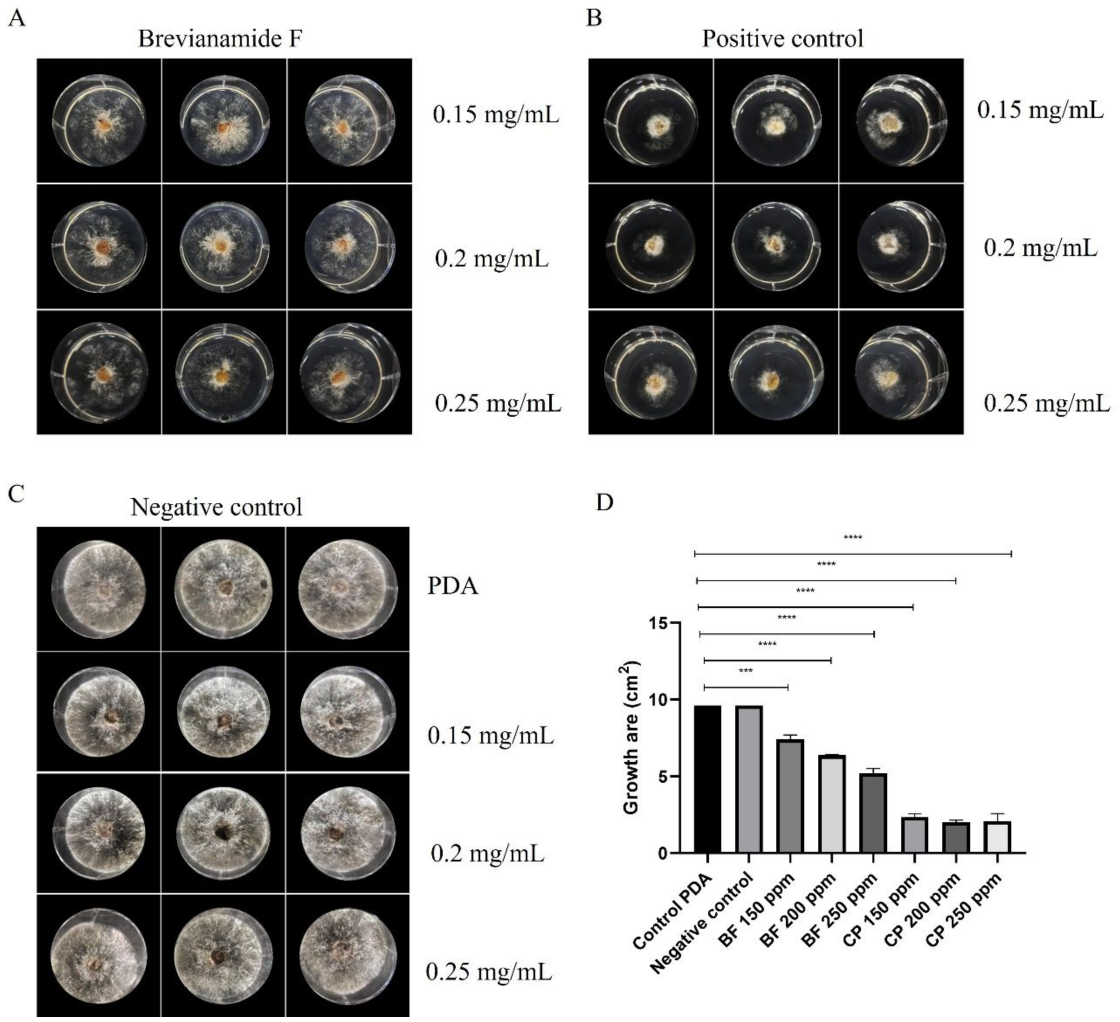

Figure S11. Minimum inhibitory concentration (MIC) assay of Brevianamide F (BF) against *Diaporthe* sp. (A) Fungal growth on PDA supplemented with BF (0.15, 0.2 and 0.25 mg/mL). (B) Positive control (commercial fungicide; 0.15, 0.2 and 0.25 mg/mL). (C) Negative control (PDA and EtOH:H_2O 1:1v:v 0.15, 0.2 and 0.25 mg/mL). (D) Growth area (cm²) after 7 days of incubation. Data represent mean ± SD (n = 3). Statistical significance was determined by one-way ANOVA with Tukey's test (***$p < 0.001$, ****$p < 0.0001$).

Supplementary File 1. ITS Sequences of Isolates Used in This Study

Sequence corresponding to *Colletotrichum* sp. isolate

>2523-1
CCTTTGTGACATAACCTATAACTGTTGCTTCGGCGGGTAGGGTCTCCGGACCCCTCC
CGGCCTCCCGCCTCCGGGCGGGTGCGGCGCCCGGCGGAGGATAACCAAACTCTGAT
TTAAACGACGTTTCTTCTGAGTGGGTACAAGCAAAATAACTAAACTTTTAAACAAC
GGATCTCTTTGGTTCTGGCACTGATGAAGAACGCAGCGAAATGGGATAAGTGAATG
TGAATTGCAGAATTCAGTGAATCATCGAATCTTTGAACGCACATTGGCGCCCACCA
GCATTCTGGCGGCGATGGCCTGTTCGAGGCTCATTTCAACCTCAAGCTCTGCTTGGT
GTTGGGGCCCTACAGCCGATGTAGGCCCTCAAAGGTAGTG

Sequence corresponding to *Diaporthe* sp. isolate

>2523-2

TTTGTGAACTTATAACCTATTGTGTTCGGCCTGGCGTCAGGCGGCCCTTCACTGAGG
CCCCCGGAGACGGGGAGCAGCCCGCCGCGGCGCCAACTAAACTCTTTGTTTCTTAA
TAGTGAATCTCGGAGTAAAAAACATAAATGGAATCAAATAACTTTCAACAACGATC
TCTTGGTTCGGCATCGATGAAGAACGCACGCAAATGGGATAAGTAATGTGAATTGC
AGAATTCAGTGAATCATCGAATCTTTGAACGCACATTGGCGCCCCTGGTAATCTCG
GGGCGGATGGCCTGTGAGGCTCATTTCAACCCTCAAGCCTGGCTTGTGTAGTGGGC
CCACTACTTCCCGGAGGTAGGCCTGAAATTCAGTGGGCGAGCTCGCCAGGACCCCG
AGGGTAGTAGTTATTCGTGGAAGGCCCTGGGGTCGGGTCCTGGCCTGTAAACCCCC
AACTCTTGAAAAATTTGACCTGCGCAGTGGAACATCCCGGTGGAACTTAAGCATAT
CAATAAGC
